# Prediction of Myasthenia Gravis Worsening: A Machine Learning Algorithm Using Wearables and Patient‐Reported Measures

**DOI:** 10.1002/acn3.70257

**Published:** 2025-11-19

**Authors:** Maike Stein, Haoqi Sun, Sophie Lehnerer, Lea Gerischer, Maximilian Mönch, Christian Meisel, Andreas Meisel, Pushpa Narayanaswami

**Affiliations:** ^1^ Department of Neurology With Experimental Neurology Charité – Universitätsmedizin Berlin, Corporate Member of Freie Universität Berlin and Humboldt‐Universität zu Berlin Berlin Germany; ^2^ Neuroscience Clinical Research Center Charité – Universitätsmedizin Berlin, Corporate Member of Freie Universität Berlin and Humboldt‐Universität zu Berlin Berlin Germany; ^3^ Berlin Institute of Health at Charité – Universitätsmedizin Berlin Berlin Germany; ^4^ Department of Neurology Beth Israel Deaconess Medical Center/Harvard Medical School Boston Massachusetts USA; ^5^ Institute of Biometry and Clinical Epidemiology Charité – Universitätsmedizin Berlin, Corporate Member of Freie Universität Berlin and Humboldt‐Universität zu Berlin Berlin Germany; ^6^ Center for Stroke Research Berlin Charité – Universitätsmedizin Berlin Berlin Germany

**Keywords:** artificial intelligence, monitoring, myasthenia gravis, remote consultation, sensors

## Abstract

**Background:**

Myasthenia gravis (MG) is a rare disorder characterized by fluctuating muscle weakness with potential life‐threatening crises. Timely interventions may be delayed by limited access to care and fragmented documentation. Our objective was to develop predictive algorithms for MG deterioration using multimodal telemedicine data.

**Methods:**

In this 12‐week randomized controlled study, 30 MG patients recorded symptoms using patient‐reported outcome measures (PROMs) and patient‐performed measures via a mobile app, alongside data from wearables. MG deterioration was defined as a ≥ 3‐point worsening in the Quantitative Myasthenia Gravis score, occurrence of MG‐related hospitalization or exacerbation. A machine learning linear classifier was trained to predict deterioration and cross‐validated. The area under the receiver operator characteristic curve (AUROC) was calculated, accepting 1–2 false alarms (FAs) per week.

**Results:**

The model achieved the best predictive performance when using all input signals (AUROC 0.85 (95% confidence interval 0.77–0.91)) and remained stable across look‐back windows of 4–10 days. Model sensitivity was 0.65 (0.48–0.83) to 0.82 (0.60–1.00) (1 and 2 FAs per week, respectively). PROMs reflected worsening symptoms before deterioration; wearables alone showed higher AUROCs.

**Interpretation:**

Multimodal self‐monitoring via MyaLink predicted MG deterioration with good performance at acceptable FA rates. This approach may enable earlier clinical interventions of MG worsening.

**Trial Registration:**

The study was registered under the German Clinical Trial Registry (DRKS00029907)

**Abbreviations**
AChR: acetylcholine receptor
AES: advanced encryption standard
app: application
CI: confidence interval
FcRn: neonatal Fc receptor
f.l.t.r.: from left to right
FVC: forced vital capacity
iMZ: integrated Myasthenia Center
iOS: iPhone Operating System
IQR: interquartile range
IVIg: intravenous immunoglobulins
LRP4: lipoprotein receptor‐related protein 4
MDD: Medical Devices Directive
MG: myasthenia gravis
MG‐ADL: Myasthenia Gravis Activities of Daily Living
MGFA: Myasthenia Gravis Foundation of America
MGFA‐PIS: MGFA Post‐Intervention Status
MG‐QoL15r: Myasthenia Gravis Quality of Life, revised version
MuSK: muscle specific kinase
PPM: patient‐performed measure
PROM: patient‐reported outcome measure
QMG: Quantitative Myasthenia Gravis score
REDCap: Research Electronic Data Capture
SBCT: single breath count test
SD: standard deviation
SSQ: Single Simple Question

## 1 Introduction

Myasthenia gravis (MG) is a rare autoimmune disorder characterized by exercise‐dependent muscle fatigability and fluctuating weakness involving ocular, bulbar, and skeletal muscles [1]. The chronic nature of MG necessitates long‐term, often lifelong, highly specialized care and individualized treatment strategies. Acute worsening of the disease (exacerbations) or myasthenic crisis (worsening requiring ventilatory support, Myasthenia Gravis Foundation of America, MGFA Clinical Class V) [2] requires urgent evaluation and treatment. Approximately 15%–20% of MG patients experience at least one myasthenic crisis during the course of the disease [3, 4]. Acute symptom worsening may indicate an impending or manifest crisis or may require treatment on its own. The ability to monitor patients and predict their occurrence could facilitate timely interventions and treatment, thereby potentially improving patient outcomes [5]. Access to clinical care, including specialists, intervals at which clinical visits are feasible, time, distance and cost constraints to travel for appointments and intersectoral information gaps can delay the detection of clinical deterioration and complicate timely interventions [6].

In recent years, digital biomarkers, defined as objectively quantifiable, technology‐derived health data [7], have emerged as a promising approach to enhance chronic disease management. By capturing continuous physiological and behavioral parameters and collecting decentralized patient‐generated health data, these technologies can complement clinical evaluations and provide personalized, ongoing support more frequently than is feasible through intermittent clinical visits [8]. In Parkinson's disease and Huntington's disease, wearable‐derived data have demonstrated good correlation with in‐clinic evaluations [9, 10], and in chronic conditions such as cancer, neuropsychiatric disorders or chronic obstructive pulmonary disease, the use of remote data has been associated with a reduced number of hospitalizations, reduced length of hospital stay and reduced number of outpatient visits [11, 12]. Despite their growing implementation in some conditions, digital biomarkers remain largely unexplored in MG. To address this gap, we have developed MyaLink, a remote monitoring application (“app”) tailored specifically for MG patients, integrating wearable sensor‐based data, patient‐reported outcome measures (PROMs) and patient‐performed measures (PPMs), detailed below.

Here, we aim to evaluate the predictive value of continuous, multipronged clinical data collected through MyaLink to predict disease worsening in MG patients using machine learning, thereby enabling the timely identification and treatment of severe deterioration.

## 2 Material and Methods

This study was approved by the ethics committee at Charité Universitätsmedizin Berlin (EA2/157/22). The study was conducted in accordance with the Declaration of Helsinki. Patients provided written consent. The study was registered under the German Clinical Trial Registry (DRKS00029907).

### 2.1 The Telemedicine Platform

**FIGURE 1:**
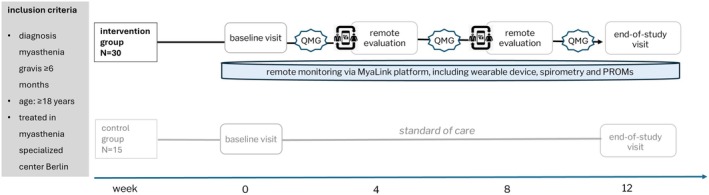
Study design. The remote measurements include (1) wearable device: SpO_2_ (peripheral blood oxygen saturation) and pulse, which were measured per minute and step count, measured continuously; (2) patient‐performed measures: Spirometry assessing forced vital capacity (FVC) measured once a week (patient could perform more frequent measurements if they felt the need to do so) and single breath count test (SBCT) measured once a week; (3) patient‐reported outcome measures (PROMs): Myasthenia Gravis Activities of Daily Living scale (MG‐ADL), Myasthenia Gravis Quality of Life 15‐item revised (MG‐QoL15r) and Single Simple Question (SSQ), measured once a week. In addition, the Quantitative Myasthenia Gravis score (QMG) was conducted throughout the study.

The telemedicine platform MyaLink, utilized in this study, was developed by Charité Universitätsmedizin Berlin and Qurasoft GmbH (software partner, Koblenz, Germany), with continuous input from a MG patient organization (German Myasthenia Association) to ensure patient‐centered design. MyaLink is a certified medical device (Medical Device Directive, MDD Class I) that complies with data protection regulations [13]. The platform consists of an application (“app”) for patients and a web‐based portal for physicians to exchange health data and a patient‐physician chat module. Patients can monitor their symptoms by completing PROMs and integrating data from external devices (Figure 1). The web‐based portal enables physicians to access patient monitoring data in real‐time, allowing therapy adjustments and patient management.

### 2.2 Patient Cohort, Eligibility Criteria

The study included adult patients (≥ 18 years) with a confirmed diagnosis of MG for ≥ 6 months, MGFA Class I‐IV [2], identified through a retrospective review of medical records at the Integrated Myasthenia Center (IMZ) at Charité Universitätsmedizin, Berlin. The diagnosis of MG was based on clinical features of fatigable muscle weakness along with one or more additional criteria: antibody positivity (acetylcholine receptor, muscle specific kinase [MuSK], low‐density lipoprotein related protein‐4 [LRP4]), electrophysiological testing (evidence of decrement on repetitive nerve stimulation/increased jitter on single fiber electromyography), and a clear response to anticholinesterase drugs. Exclusion criteria were the presence of psychiatric or neurological disorders that could affect their report of symptoms or their ability to use Myalink. Patients were randomized in a 2:1 ratio into an intervention group and a control group using a digital randomization tool [14], stratified by sex and MGFA clinical class at study entry.

### 2.3 Study Design

The study design has been previously described (Figure 1) [13]. Briefly, this study included 45 MG patients who were followed for a 12‐week period between April and September 2023. The control group received standard care, while the intervention group received standard care plus remote monitoring with MyaLink. As the focus of this analysis is the remote monitoring data to develop predictive models, the control group will not be discussed further. Standard care involved two comprehensive study center visits, baseline and end of the study, which included a regular clinical appointment, clinical evaluations, a MG‐specific history, assessment of comorbidities, medications, hospitalizations, and self‐reported exacerbations. Both groups completed PROMs and PPMs and underwent MG‐specific physical examinations (QMG) [15]. Patients in the intervention group used the MyaLink app and were equipped with an activity tracker (Garmin Vivosmart 5) and a digital spirometer (MIR Spirobank Smart). The PROMs were reported weekly and included Myasthenia Gravis Activities of Daily Living (MG‐ADL, 0–24 points, higher score represents worse ADL) [16], Myasthenia Gravis Quality of Life, 15 item, revised (MG‐QoL15r, 0–30 points, higher score represents worse QOL) [17] and Single Simple Question (SSQ, 0%–100%, higher % represents better perceived health status) [18]. The PPMs (spirometry and single breath count test, SBCT) were performed weekly (Table 1). Patients were trained prior to the study to perform these measures. Reminders were delivered through the app whenever a patient was assigned a questionnaire or spirometry measurement. Patients could perform spirometry more frequently, but the other measures could only be performed at prespecified intervals. If patients had MG‐related hospitalizations during the study, additional QMG evaluations were obtained if feasible. Wearable data were collected either continuously (step count) or at 1‐min intervals (heart rate and oxygen saturation [SpO_2_]). At weeks 4 and 8, the study physicians remotely reviewed all monitoring data in the web‐based portal and initiated patient contact via the chat function if needed (e.g., for medication adjustments). All study‐related data, including baseline and end of study visit, were recorded in REDCap (Version 13.7.31).

**TABLE 1:** Data categories, their respective features, frequency, and device of assessment.

Data category	Feature	Frequency, visit	Device
CRO	QMG (Quantitative Myasthenia Gravis score)	BL, EOS Further assessments are possible during MG‐related hospitalizations during the study	n/a
	MGFA‐PIS (Post‐Intervention Status)	EOS	
PROM	MG‐ADL (Activities of Daily Living)	Weekly, BL, EOS	
	MG‐QoL15r (Quality of Life)	Weekly, BL, EOS	n/a
	SSQ (Single Simple Question)	Weekly, BL, EOS	
PPM	SBCT (single breath count test)	Weekly, BL, EOS	n/a
	FVC (forced vital capacity)	Weekly, BL, EOS	MIR Spirosmart One
Exacerbations	Number, triggers, symptoms	EOS	n/a
MG‐related hospitalizations	Number of MG‐related hospitalizations, duration, level of care, triggers, treatment	BL, EOS	n/a
Wearable (vital parameters)	Physical activity (step count)	Continuously	Garmin Vivosmart 5
	heart rate	1/min	Garmin Vivosmart 5
	Peripheral oxygen saturation (SpO_2_)	1/min	Garmin Vivosmart 5

Abbreviations: BL, baseline visit; CRO, clinician‐reported outcome; EoS, end of study visit; MG, myasthenia gravis; PPM, patient‐performed measure; PROM, patient‐reported outcome measure.

### 2.4 Definition of MG Deterioration

MG deterioration and their time points were defined as follows: (1) QMG‐defined: Date of ≥ 3 point increase in QMG score between two measurements (excluding QMG ocular items); (2) Date of any MG‐related hospitalization (excluding hospitalization for surgery or elective hospitalizations, e.g., for infusions); and (3) Self‐reported exacerbations: At the end‐of‐study visit, patients were asked to retrospectively report any exacerbations during the study period, and this was validated for plausibility by the physician. During analysis, the timing of the exacerbation was reconstructed as precisely as possible using all available clinical data, including chat records. The date was defined as the day after the chat message indicating worsening was received, unless the patient specified the date. Only those exacerbations for which a reliable time point could be established were included in the model.

### 2.5 Data Preprocessing

We converted the per‐minute measurements from wearable devices to average daily measurements for each day. For SpO_2_, we used values when pulse rates were also available, since pulse is more stable and less prone to technical interference than SpO_2_. We excluded plateau periods, defined as periods ≥ 1 h with unchanged SpO_2_, which were likely artifacts. We excluded step counts when the patient was in a wheelchair. One patient exhibited abnormally high step counts during a specific period, which was excluded (during this period, steps were also recorded via an internal mobile health app integrated into MyaLink, leading to overestimation of step counts).

### 2.6 Model Training, Validation, and Interpretation

**FIGURE 2:**
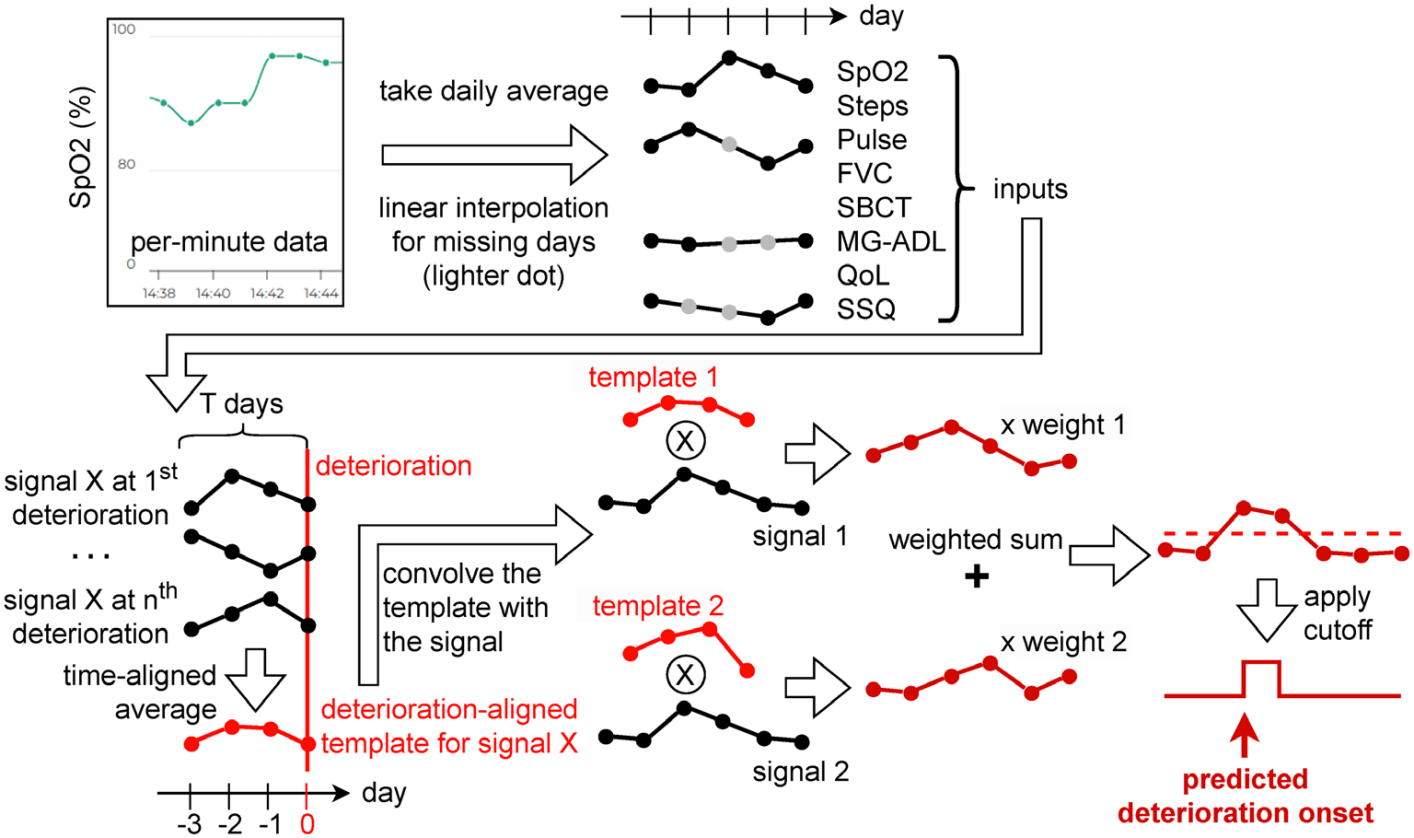
A flowchart to illustrate the data processing steps. First, the preprocessed data are converted into daily averages. Linear interpolation was used to fill the missing values on days when data were not available. We then obtained the T‐day average of each input signal, time‐aligned to the deterioration onsets (for all subtypes), which we refer to as templates. T is an adjustable look‐back period. The template of each input signal then underwent a convolution‐like operation (circled cross operator), where the templates were slid over the input signal at a daily resolution to obtain a time series of Pearson's correlations (*R*, ranges from −1 to +1), and then transformed into a time series of similarities that ranged from 0 to 1 by (*R* + 1)/2. The model output was obtained by a weighted sum of similarities weighted by the model coefficients, i.e., input signal importances. Finally, the output was binarized using a cutoff value corresponding to a specific false alarm rate. A higher cutoff corresponds to a lower false alarm rate. FVC, forced vital capacity; MG‐ADL, Myasthenia Gravis Activities of Daily Living; QoL, Myasthenia Gravis Quality of Life‐15 item, revised version; SBCT, single breath count test; SpO_2_, oxygen saturation; SSQ, Single Simple Question.

The data processing steps are shown in Figure 2. The data were analyzed retrospectively. The signals were z‐transformed based on individual‐specific mean and standard deviation. For every signal, we computed a signal‐baseline‐adjusted template to describe the average dynamics before deterioration: first, an individual‐level signal baseline was obtained as the average value from study start to 1 week before the first deterioration or 1 month into the study, whichever was earlier; then the template was obtained by subtracting this baseline from the signal and aligning all signals to a specific look‐back period before the deterioration onset. Next, each input signal was transformed using a convolution‐like operation, i.e., we slid the template over the signal to compute a time series of Pearson's correlations (transformed to [0, 1] by (*R* + 1)/2), representing the similarity between the current signal segment and the average dynamics before deterioration. Therefore, for prospectively new participants, the z‐transformation above is optional even when the individual‐specific mean and standard deviation are unknown, due to the invariance of Pearson's correlation. We then defined a linear machine learning classifier to perform binary classification using these time series of similarities as inputs. The classification target was set to 1 on days within a 7‐day window before deterioration (to accommodate timing uncertainty) and 0 otherwise. The 7‐day window ensured the model's predictive ability before the onset of deterioration. The model was trained to maximize the effect size (Cohen's *d*) of the model outputs between deterioration and non‐deterioration periods. During model training, the signal‐wise model coefficients (i.e., input signal importance) were constrained to be nonnegative and have a unit mean to enhance model interpretability and ensure the output remains within the range of [0, 1]. Finally, the model output was interpreted as a probability that increases when a patient's current signal patterns resemble the pre‐deterioration templates.

To validate the model, we used leave‐one‐subject‐out cross‐validation (LOSO‐CV) to ensure subject‐independent generalization. For each fold, the model was trained on all subjects except one and tested on the held‐out subject. The model probability outputs were combined across folds and evaluated using receiver operating characteristic (ROC) curves. By varying thresholds for dichotomizing the model probability output, the *x*‐axis of the ROC represents the false alarm (FA, model‐predicted deterioration in the absence of true clinical deterioration as defined above) rate (per week, maximum 7 per week) at a daily level, and the *y*‐axis represents sensitivity (true positive rate, maximum 1) at an event level; therefore the best possible area under the ROC curve (AUROC) is 7. For convenience, the AUROC can be divided by 7 to obtain the common AUROC scale between 0 and 1. We examined the sensitivities at 1 and 2 false alarms per week for simple interpretation. To assess the contribution of different input modalities, separate models were trained using (1) wearable sensors (SpO_2_, steps, pulse), (2) PPM (FVC, SBCT), (3) PROMs (MG‐ADL, MG‐QoL15r, SSQ), and (4) all modalities, 1–3 (Table 1). Lastly, model interpretation was performed by plotting the templates and the average signal importances across the LOSO CV folds.

We also used a random predictor model as the null model for comparison as a baseline. The random predictor assumed a Bernoulli distribution at each day, where the positive probability was set to the actual deterioration proportion (including the pre‐deterioration 7‐day window) among all days in the training set. The same LOSO CV was performed. The same performance metrics were used.

## 3 Results

### 3.1 Cohort Characteristics

As shown in Table 2, the final cohort consisted of 30 participants. The median age was 48 years, and 70% were female. The median disease duration was about 5 years. Most patients had mild to moderate disease (MGFA Class II–III). Over half were receiving immunosuppressive or immunomodulatory therapies. Five patients were hospitalized, for a total of eight MG‐related hospitalizations (Table 3).

**TABLE 2:** Patient characteristics at baseline.

	Participants (*N* = 30)
Age, in years	
Median [IQR; min–max]	47.5 [40.5, 57.5; 23–82]
Disease duration, in years	
Median [IQR; min–max]	5.5 [2.0, 9.8; 1–17]
Sex/Gender^a^, *n* (%)	
Female	21 (70.0)
Male	9 (30.0)
MGFA clinical class, *n* (%)	
I	1 (3.3)
II	11 (36.7)
III	13 (43.3)
IV	5 (16.7)
Thymectomy, *n* (%)	21 (70.0)
Thymoma^b^, *n* (%)	2 (9.5)
Antibody status, *n* (%) (multiple answers possible)	
AChR	20 (66.7)
MuSK	1 (3.3)
LRP4	2 (6.7)
Seronegative	7 (23.3)
Of these, multiple antibodies, *n* (%)	
AChR and LRP4	1 (3.3)
Therapy (at baseline), *n* (%) (multiple answers possible)	
Symptomatic therapy	28 (93.3)
Glucocorticoids	18 (60.0)
Conventional immunosuppressive therapies^c^	17 (56.7)
Other^d^	17 (56.7)
IVIg, PLEX, Efgartigimod, Eculizumab, Ravulizumab, Rituximab	
Baseline MG‐ADL	
Median [IQR; min–max]	10.0 [5.3, 11.0; 1–14]
Baseline MG‐QoL15r	
Median [IQR; min–max]	15.5 [12.3, 20.8; 4–28]
Baseline QMG	
Median [IQR; min–max]	15.0 [12.3, 17.0; 1–25]
Baseline FVC, [liters, %]	
Liters [IQR], %	3.4 [2.7, 3.7], 93.5%

Abbreviations: AChR, acetylcholine receptor; FVC, forced vital capacity; IVIg, intravenous immunoglobulin; LRP4, lipoprotein receptor‐related protein 4; MG‐ADL15, Myasthenia Gravis Activities of Daily Living; MGFA, Myasthenia Gravis Foundation of America; MG‐QoL15r, Myasthenia Gravis Quality of Life‐15 item, revised version; MuSK, muscle specific kinase; PLEX, plasma exchange; QMG, Quantitative Myasthenia Gravis score.

^a^ Both categories were obtained at the baseline visit, no discrepancies between sex/gender for any patient.

^b^ *n* (%) with respect to patients with thymectomy.

^c^ Azathioprine, mycophenolate mofetil, methotrexate.

^d^ In the last 12 months; multiple treatments at the same time possible, e.g., continuation of rituximab treatment and need for rescue therapy with PLEX.

**TABLE 3:** Deteriorations (hospitalizations and self‐reported exacerbations).

	Participants (*N* = 29^a^)
Number of patients with MG exacerbation(s) or MG‐related hospitalization(s), *n* (%)	15 (51.7)
Number of patients with exacerbation(s), *n* (%)	13 (44.8)
Number of hospitalized patients (MG‐related hospitalization(s)), *n* (%)	5 (17.2)
MGFA clinical class (baseline)^b^, *n* (%)	
III	2 (40.0)
IV	3 (60.0)
Total number of hospitalizations	8
Type of ward^c^ (multiple answers possible)	
Intensive care unit	1 (12.5)
Non‐intensive care setting	8 (100.0)
Therapy^c^ (multiple answers possible)	
IVIg	4 (50.0)
Plasmapheresis/immunoadsorption	1 (12.5)
Supportive therapy^c^	
Noninvasive ventilation (NIV)	1 (12.5)

Abbreviations: IVIg, intravenous immunoglobulin; MG, myasthenia gravis; MGFA, Myasthenia Gravis Foundation of America.

^a^ One patient had incomplete follow‐up (dropout).

^b^ *n* (%) relative to the total number of hospitalized patients.

^c^ *n* (%) relative to the total number of hospitalizations.

### 3.2 MG Deterioration Prediction Performance

**FIGURE 3:**
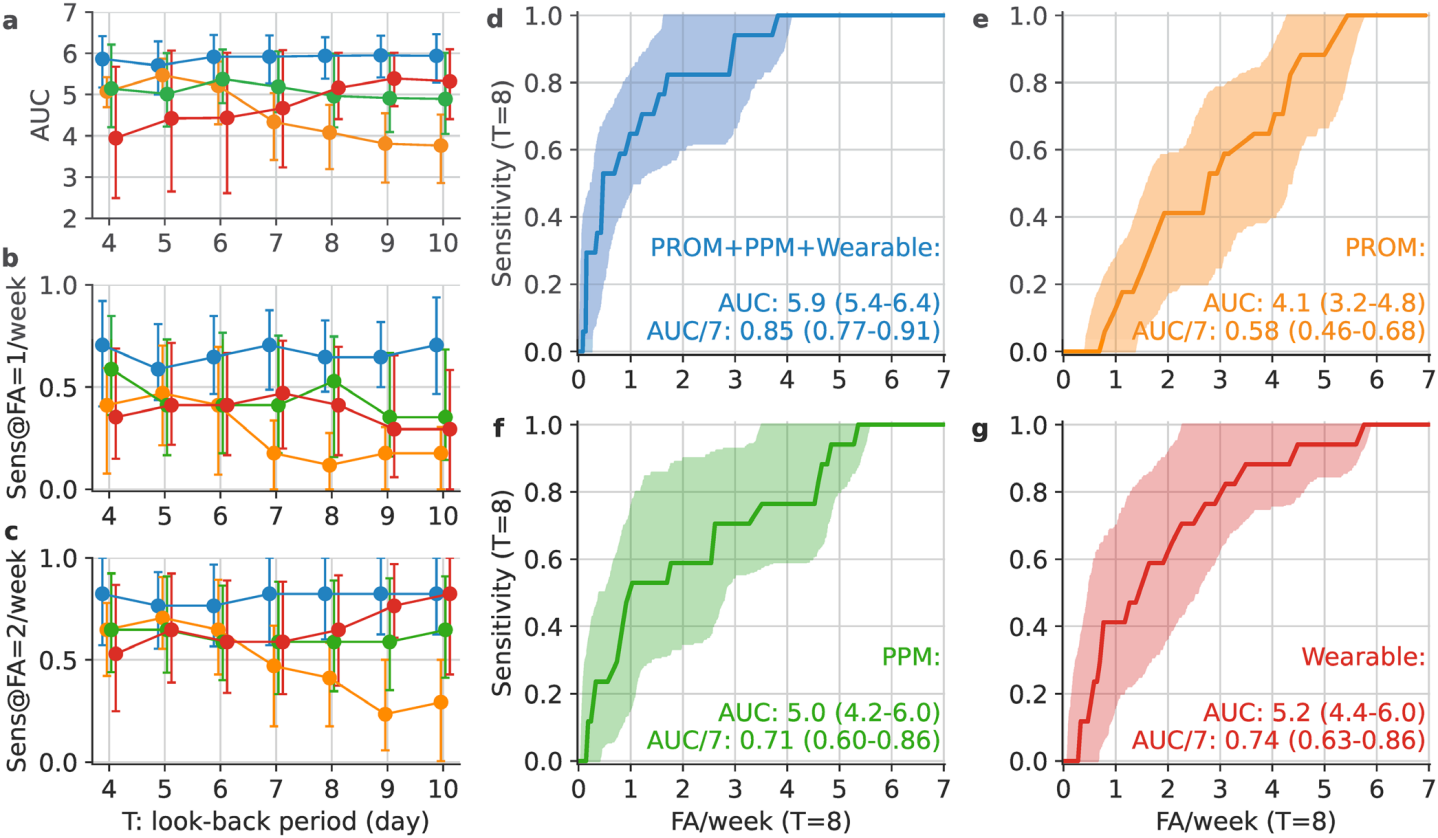
Multivariate AUROC (area under the receiver operating characteristic curve) (a), sensitivities at 1 (b) and 2 (c) false alarms (FA) per week at look‐back periods T = 4–10 days. The four plots on the right‐hand side (d–g) show the ROC (receiver operating characteristic) curves using different input signals at T = 8. Patient‐performed measures (PPMs): forced vital capacity (FVC), single breath count test (SBCT); wearable: peripheral oxygen saturation (SpO_2_), steps, pulse; patient‐reported outcome measures (PROMs): Myasthenia Gravis Activities of Daily Living (MG‐ADL), Myasthenia Gravis Quality of Life, revised version (MG‐QoL15r), Single Simple Question (SSQ).

Signals from wearable devices achieved the highest AUROC in univariate analysis (Table S1) with 6.3 (95% confidence interval, CI: 5.7–6.8) for steps and 6.1 (CI: 5.6–6.6) for SpO_2_, outperforming the best multivariate performance based on PROM + PPMs + wearables with 5.9 (CI: 5.4–6.4). As a baseline performance, the random predictor of deterioration obtained an AUROC of 4.1 (CI: 3.5–4.9), which was significantly lower than the model performances. Figure 3 shows the multivariate AUROCs and sensitivities at 1 and 2 false alarms (FA) per week at different look‐back periods (T, in days prior to the deterioration date). The AUROC using all input signals was consistently higher than the AUROC using a subset of the input signals across 4–10 days of look‐back periods. For example, with a look‐back period of T = 8 days, the AUROC was 5.9 (CI: 5.4–6.4, 0.85 when divided by 7 to the common scale between 0 and 1) using all signal inputs (Figure 3d, Table S1). Using PPMs only resulted in stable AUROCs of around 5.0 (Figure 3f, Table S1). When comparing PROMs only vs. wearables only, the AUROC was higher using PROMs at shorter look‐back periods and higher using wearables at longer look‐back periods (Figure 3a). The alternative of using PROMs and wearables resulted in stable performance when using all input signals. Given the higher assessment frequency of wearable signals (averaged to daily) compared to PPMs and PROMs (weekly), we investigated whether reducing the assessment frequency of wearable signals would compromise performance. As shown in Figure S1, the AUROC and sensitivities at assessment intervals longer than 1 day were indeed lower. The sensitivities at 1 and 2 false alarms per week showed similar trends to those of AUROC (Figure 3b,c).

### 3.3 Model Interpretation

**FIGURE 4:**
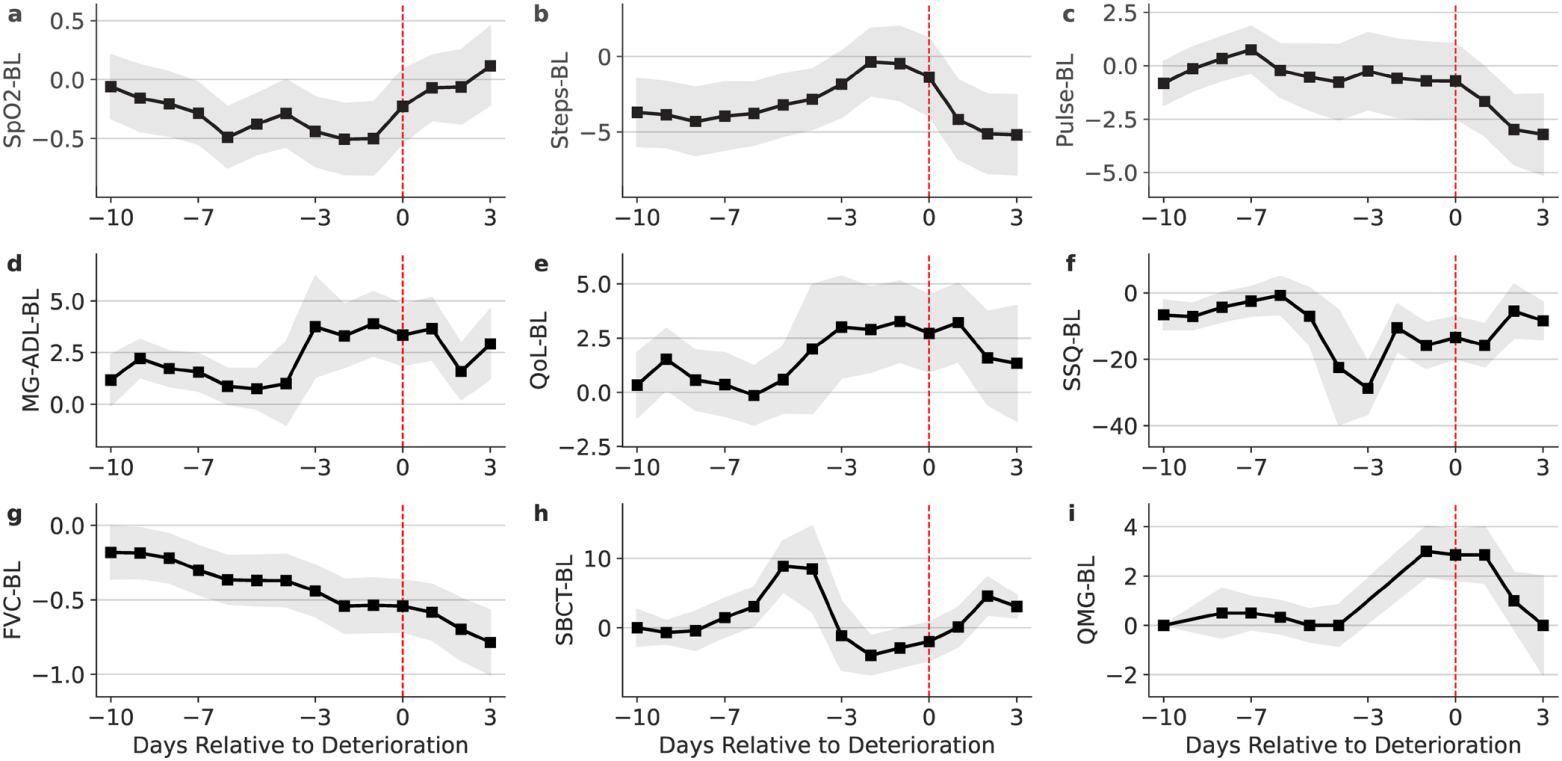
The average input signals and Quantitative Myasthenia Gravis score (QMG), subtracted from signal baseline (BL), and then time‐aligned to the onset of deterioration (vertical dashed red line), from 10 days before to 3 days after the onset. The *x*‐axis represents the days relative to the onset of deterioration. The black lines represent the averaged signals, which serve as templates for convolving with the corresponding signals. The gray shading is the 95% confidence interval. BL, signal baseline; FVC, forced vital capacity; MG‐ADL, Myasthenia Gravis Activities of Daily Living scale; QoL, Myasthenia Gravis Quality of Life 15‐item revised; SBCT, single breath count test; SpO_2_, peripheral oxygen saturation; SSQ, Single Simple Question.

Figure 4 shows the average signal‐baseline‐adjusted templates from 10 days before to 3 days after the deterioration onset. For the wearables, SpO_2_ reached its minimum about 1–2 days before the onset; step count was higher about 2 days before the onset of the deterioration, but declined subsequently, and pulse rate was higher about 3 days prior to the onset and then dropped. For the PPMs, FVC showed a clear decreasing pattern in the entire span from 10 days before the onset; SBCT reached its minimum about 2 days before the onset. For the PROMs, MG‐ADL and MG‐QoL15r showed higher scores about 3 days before the onset and remained high for 1 day after the onset; SSQ reached its minimum about 3 days before the onset. The QMG showed a clear increase before the onset. Overall, changes were noted about 2–3 days before the onset of deterioration, except for FVC, which showed a decline as long as 10 days before the onset of deterioration.

**FIGURE 5:**
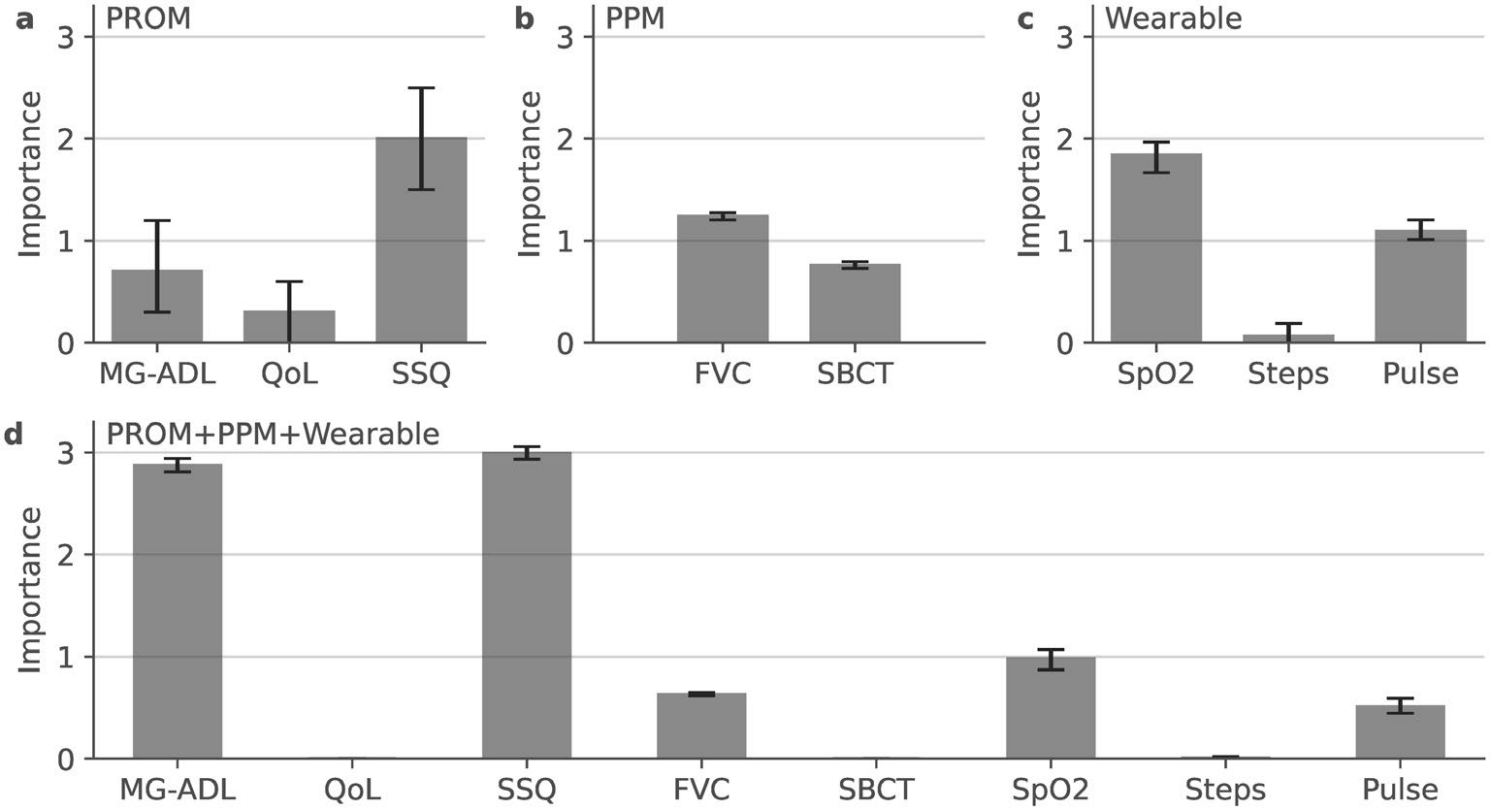
The signal importances that indicate the relative importance of input signals that were combined to make the prediction. A higher signal importance indicates that it is more important for the prediction. The signal importances were obtained by averaging the values across all cross‐validation folds. The error bars are the 95% confidence intervals from bootstrapping 1000 times. FVC, forced vital capacity; MG‐ADL, Myasthenia Gravis Activities of Daily Living scale; MG‐QoL15r, Myasthenia Gravis Quality of Life 15‐item revised; PPM, patient‐performed measure; PROM, patient‐related outcome measure; SBCT, single breath count test; SpO_2_, peripheral oxygen saturation; SSQ, Single Simple Question.

Figure 5 shows the signal importance of each input signal when combined to make a prediction. Due to the constraint of being nonnegative, some values become exactly zero, and the signals with zero importance are excluded. The relative importance of input signals was maintained when using all signals compared to a subset. Among the PROMs, MG‐ADL and SSQ were more important than MG‐QoL15r in predicting the onset of MG deterioration. Among the PPMs, FVC was more important than SBCT. Among the wearables, SpO_2_ and pulse rate were more important than step counts.

## 4 Discussion

In this study, we developed and tested a machine learning‐based model for predicting deterioration in MG clinical status using wearable data and self‐reported measures (PROMs and PPMs). The model demonstrated moderate overall predictive performance (AUROC of 0.85; CI: 0.77–0.91 and sensitivity of 0.82; CI: 0.60–1.00) with the best results achieved when integrating data from all sources, accepting 2 false alarms per patient per week. With 1 false alarm per week, the sensitivity was 0.65 (CI: 0.48–0.83). The model's prediction window remained stable across a 4‐to‐10‐day timeframe before deterioration, indicating a potential intervention window.

### 4.1 Model Performance and Predictive Accuracy

The model's performance in terms of sensitivity for predicting MG deterioration was moderate, with some variability based on the type and combinations of input data. The highest predictive accuracy was obtained by integrating multiple types of input signals, including wearable data, PPMs and PROMs, emphasizing the importance of multisource data integration in telemedicine applications such as MyaLink, which could complement or potentially substitute for in‐clinic assessments. The observed differences between PROMs and wearable data in prediction performance across look‐back periods may be attributed to the varying data collection intervals for each signal. When using PROMs, prediction performance was higher with shorter look‐back periods of 7 days and decreased with longer look‐back periods, as the data was assessed weekly, making prior data less reflective of the patient's current state. In contrast, models based on wearable data, which were collected continuously, showed better performance with longer look‐back periods, as longer time windows allowed for clearer patterns to emerge. Our observation is consistent with studies in other neurological disorders like multiple sclerosis, Huntington's disease and Friedreich's ataxia, where wearable‐derived data correlated well with clinical evaluations [19, 20, 21].

Allowing for 2 false alarms per week improved the sensitivity of predicting MG deterioration. The acceptance threshold for false alarms is crucial for optimizing sensitivity, which is necessary to avoid missing a deterioration that requires urgent management. However, false alarms also have negative clinical consequences, such as recommendations for emergency evaluation. The degree of such “oversensitivity” that is acceptable needs to be defined. It may also be different between patients, i.e., in poorly controlled MG with frequent exacerbations, it may be acceptable to “set” the model at a higher false alarm rate, compared to reasonably controlled MG. Because a missed deterioration has serious consequences, this trade‐off should be carefully evaluated in future refinements of the model. Evaluating and addressing potential sources of false alarms will be a focus of future refinements.

The model's predictive performance remained relatively stable within a 4–10‐day window before a deterioration, providing a sufficient timeframe for urgent clinical intervention. However, the model may not effectively capture small incremental changes within this period due to unknown thresholds or a possible ceiling effect. We did not evaluate look‐back periods greater than 10 days, given our small sample size, and such analysis may be informative. Interestingly, transient increases in step count and pulse rate were observed prior to deterioration. This may reflect compensatory overexertion before the onset of overt fatigability or early autonomic imbalance during subclinical decline. Given the gradual nature of MG worsening and the use of daily averaged wearable data, these signals may miss small incremental changes not represented across days missing early markers of decline in the last days before deterioration onset.

### 4.2 Factors That May Have Influenced Prediction Performance

Our machine learning‐based algorithm did not account for interindividual differences such as disease severity, personality traits, or anxiety levels, all of which may influence symptom reporting and wearable‐derived data. The duration and severity of the disease may play a role, as patients may be more anxious earlier in the disease and with more severe symptoms. Adjustment for these factors may improve performance‐predictive ability. Recent studies have emphasized the importance of considering these factors in interpreting patient‐generated health data [22]. Furthermore, variations in treatment regimens, including medication changes were not explicitly modeled. Prior training in using the devices may impact data quality and model performance needs to be evaluated before and after training. Standardizing the data that patients are trained to recognize as signs of deterioration may help reduce false alarms and improve data reliability. Wearable data was prone to noise, and identifying, pilot testing, and validating the most reliable wearables would also improve data quality.

### 4.3 Feature Analysis and Data Practicality

Feature importance analysis identified MG‐ADL, SSQ, SpO_2_ and FVC as key predictors (Figure 5). Additionally, artificially reducing the wearable data assessment interval to match the lower frequency of PROMs and PPMs resulted in a decline in sensitivity (Figure S1), reinforcing the need for frequent data collection to optimize model performance and detect subtle deterioration patterns. While frequent assessments enhance prediction accuracy, they also increase patient burden, particularly for actively collected data such as PROMs and PPMs.

### 4.4 Limitations

The study has several limitations, including a relatively small sample size and a short observation period, which may impact the model's generalizability. Additionally, the retrospective identification of the time point of self‐reported exacerbations could have limited temporal precision and influenced time‐sensitive analyses. Selection bias with a higher likelihood of recruiting patients familiar with digital devices cannot be excluded. The study also relied on linear interpolation for larger or unequal assessment intervals, a simplification that does not fully capture the complexity of disease progression. Future research should explore alternative modeling approaches, such as deep learning methods, to better capture nonlinear trends in the data. Lastly, the templates resembling pre‐deterioration dynamics were averaged across all patients, potentially leading to an overestimation of performance, although mitigated by the LOSO cross‐validation.

### 4.5 Clinical Implications

This study highlights the potential of remote patient monitoring to facilitate the early detection of MG deterioration. This is likely to be of value for patients with severe or unstable MG, those in rural areas or with limited access to centers specialized in MG management and those with limited capacity for travel. By providing real‐time data, telemedicine platforms could enhance clinical decision‐making and facilitate timely interventions. However, caution is warranted when interpreting the results due to the model's moderate sensitivity, potential for false‐positive alarms, and technical limitations, necessitating comprehensive data preprocessing. Remote monitoring solutions may also support interdisciplinary care coordination and enhance patient engagement, potentially leading to improved health outcomes. However, generalizability may be limited by factors such as basic digital literacy among users.

While our findings suggest that telemedicine‐based predictive models hold promise for detecting MG deterioration, further refinements are needed before their widespread clinical adoption. Addressing data consistency, feature selection, and the balance between sensitivity and specificity will be critical in future iterations of the model. Factors such as patient characteristics, disease variability which may affect the false alarm rate that is acceptable, and inconsistencies in data collection also need to be considered.

## Author Contributions

Conceptualization: M.S., H.S., C.M., and P.N. Methodology: M.S., H.S., and P.N. Investigation: M.S., S.L. Resources: A.M. and P.N. Data curation: M.S., H.S., M.M., and P.N. Writing – original draft: M.S., H.S., and P.N. Writing – review and editing: S.L., L.G., M.M., C.M., and A.M. Formal analysis: H.S. Visualization: M.S. and H.S. Supervision: P.N. Project administration: M.S. and P.N. Funding acquisition: M.S. and P.N.

## Conflicts of Interest

M.S. has received speaker's honoraria and honoraria for attendance at advisory boards from Argenx and Alexion. S.L. has received honoraria for attending advisory boards and speeches from Alexion, AstraZeneca Rare Disease, Argenx, Biogen, Hormosan, Huma, Johnson & Johnson, Merck, Roche, and UCB. L.G. has received speaker's honoraria from Alexion and Roche. M.S., S.L., and L.G. are the co‐founders and shareholders of RareLink digital health GmbH. A.M. received speaker's honoraria, served as an advisor board/DSMB member and consultant, and has received research grants (paid to his institution) from Alexion AstraZeneca Rare Disease, Amgen, Argenx, Axunio, Desitin, Genpharm, Grifols, Hormosan, Immunovant, Johnson & Johnson, Merck, Novartis, Octapharma, Regeneron, Sanofi and UCB. He is chairman of the *Association for Research of Myasthenic Syndromes in Germany* and a member of the medical advisory board of the German Myasthenia Gravis Society. P.N. has received grant support from NIH, PCORI and Alexion. P.N. has served as a consultant/advisory board member for Alexion, Amgen, Argenx, Cartesian, CVS Caremark, Dianthus, Immune Abs, Immunovant, Johnson and Johnson, Serono‐EMD, UCB and Viridian. DSMB: on the Sanofi, Argenx, and NMD pharma. Royalties: Springer Nature. All other authors have no conflicts of interest to report.

## Supporting information


**Table S1:** Univariate and multivariate AUC and sensitivities.
**Figure S1:** Assessment interval versus performance using univariate wearable signal input. The performances were assessed using a look‐back period (T) of 8 days.
**Figure S2:** Visualization of all cases. The top panel shows the prediction: black curve as the raw prediction, brown binary curve as the binarized prediction, and the horizontal brown dashed line as the threshold for binarization. The vertical lines are deteriorations, with a 7‐day window before the exact day. Red vertical line and window indicate Quantitative Myasthenia gravis score (QMG)‐based deterioration; purple vertical line and window indicate self‐reported deterioration; and cyan vertical line and window indicate hospitalization‐based deterioration. The lower panels indicate the actual signals. Each marker dot represents 1 day. Days without signal are connected using lines.

## Data Availability

The data that support the findings of this study are available from the corresponding author upon reasonable request.
