# Supplementary material for: Prediction of Myasthenia Gravis Worsening: A Machine Learning Algorithm Using Wearables and Patient‐Reported Measures
Source: Ann Clin Transl Neurol. 2025 Nov 19;13(4):714–23. doi: 10.1002/acn3.70257 (PMC13071130; doi:10.1002/acn3.70257)

# **Supplementary material**

**Tables**

**Table S1.** Univariate and multivariate AUC and sensitivities.

| **Category** | **Name** | **AUC** | **Sensitivity@FA=1/week** | **Sensitivity@FA=2/week** |
| --- | --- | --- | --- | --- |
| Univariate | SpO2 | 6.1 (5.6-6.6) | 0.71 (0.50-0.93) | 0.88 (0.76-1.00) |
|  | Step | 6.3 (5.7-6.8) | 0.83 (0.57-1.00) | 0.92 (0.67-1.00) |
|  | Pulse | 5.4 (4.5-6.1) | 0.41 (0.17-0.71) | 0.82 (0.58-1.00) |
|  | FVC | 5.4 (4.9-6.1) | 0.47 (0.30-0.75) | 0.71 (0.55-0.90) |
|  | SBCT | 5.1 (4.2-6.4) | 0.62 (0.35-0.90) | 0.69 (0.40-0.98) |
|  | MG-ADL | 3.8 (2.5-4.9) | 0.29 (0.10-0.50) | 0.35 (0.11-0.56) |
|  | MG-QoL15r | 4.5 (3.8-5.3) | 0.35 (0.15-0.57) | 0.41 (0.17-0.68) |
|  | SSQ | 5.4 (4.7-6.2) | 0.47 (0.34-0.86) | 0.76 (0.58-0.93) |
| Multivariate | PROM+PPM+Wearable | 5.9 (5.4-6.4) | 0.65 (0.48-0.83) | 0.82 (0.60-1.00) |
|  | PROM | 4.1 (3.2-4.8) | 0.12 (0.00-0.28) | 0.41 (0.18-0.58) |
|  | PPM | 5.0 (4.2-6.0) | 0.53 (0.16-0.75) | 0.59 (0.35-0.89) |
|  | Wearable | 5.2 (4.4-6.0) | 0.41 (0.17-0.70) | 0.65 (0.37-0.91) |

AUC - Area Under the Curve; FA – false alarm; sPO2 – peripheral oxygen saturation; FVC – Forced Vital Capacity; SBCT – Single Breath Count Test; MG-ADL – Myasthenia gravis Activities of Daily Living; MG-QoL15r – Myasthenia gravis Quality of Life, revised version; SSQ – Single Simple Question; PROM – patient-related outcome measure; PPM – patient-performed measure

**Figures**

**
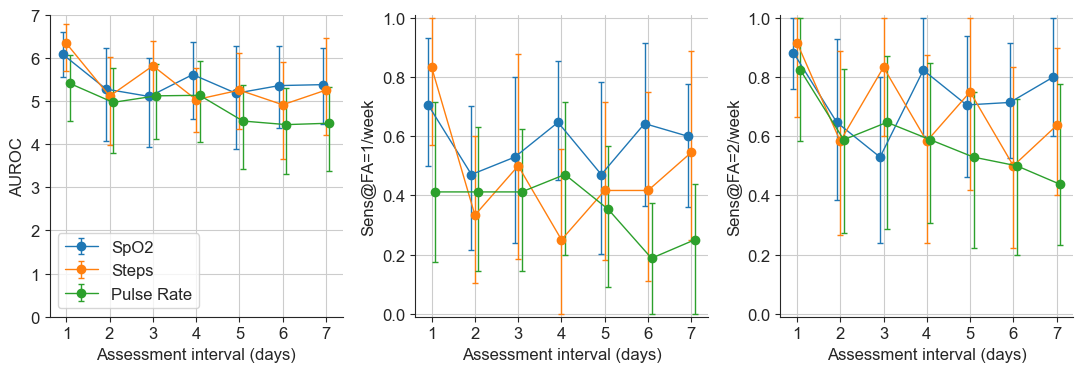
**

**Figure S1.** Assessment interval vs. performance using univariate wearable signal input. The performances were assessed using a look-back period (T) of 8 days.

AUC - Area Under the Curve; FA – false alarm; sPO2 – peripheral oxygen saturation

**Figure S2.** Visualization of all cases. The top panel shows the prediction: black curve as the raw prediction, brown binary curve as the binarized prediction, and the horizontal brown dashed line as the threshold for binarization. The vertical lines are deteriorations, with a 7-day window before the exact day. Red vertical line and window indicate Quantitative Myasthenia gravis score (QMG)-based deterioration; purple vertical line and window indicate self-reported deterioration; and cyan vertical line and window indicate hospitalization-based deterioration. The lower panels indicate the actual signals. Each marker dot represents one day. Days without signal are connected using lines.

sPO2 – peripheral oxygen saturation; FVC – Forced Vital Capacity; SBCT – Single Breath Count Test; MG-ADL – Myasthenia gravis Activities of Daily Living; QoL – Myasthenia gravis Quality of Life- 15 item, revised version; SSQ – Single Simple Question


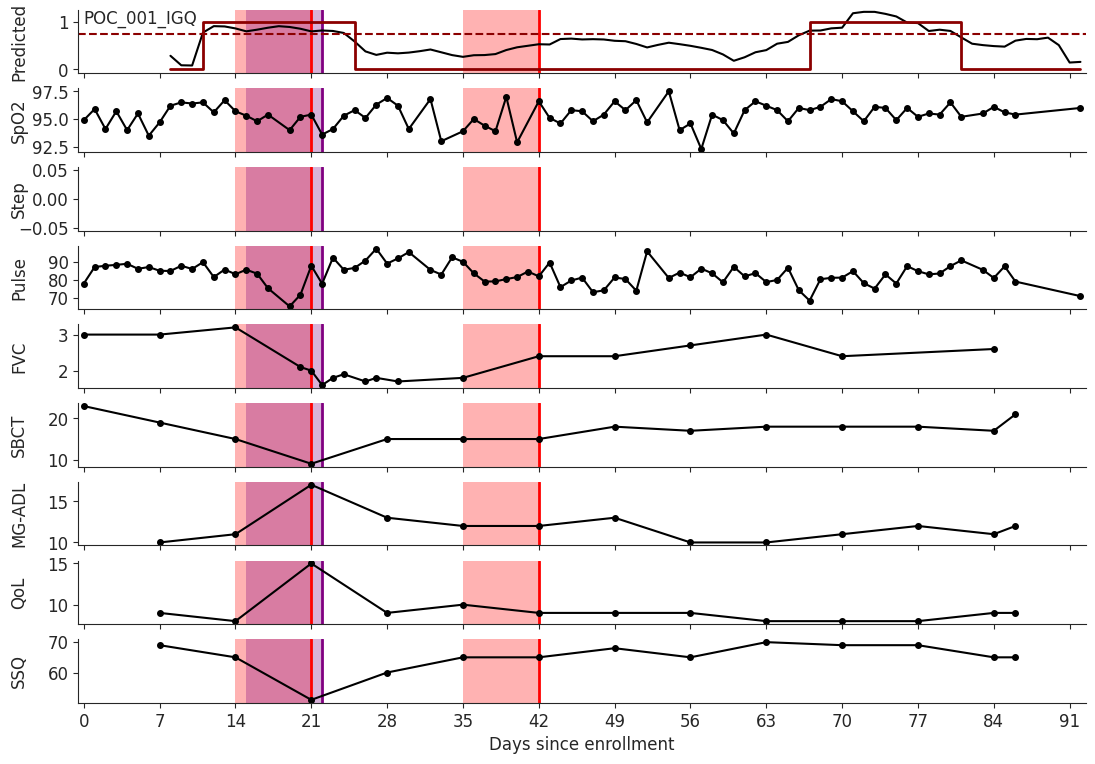


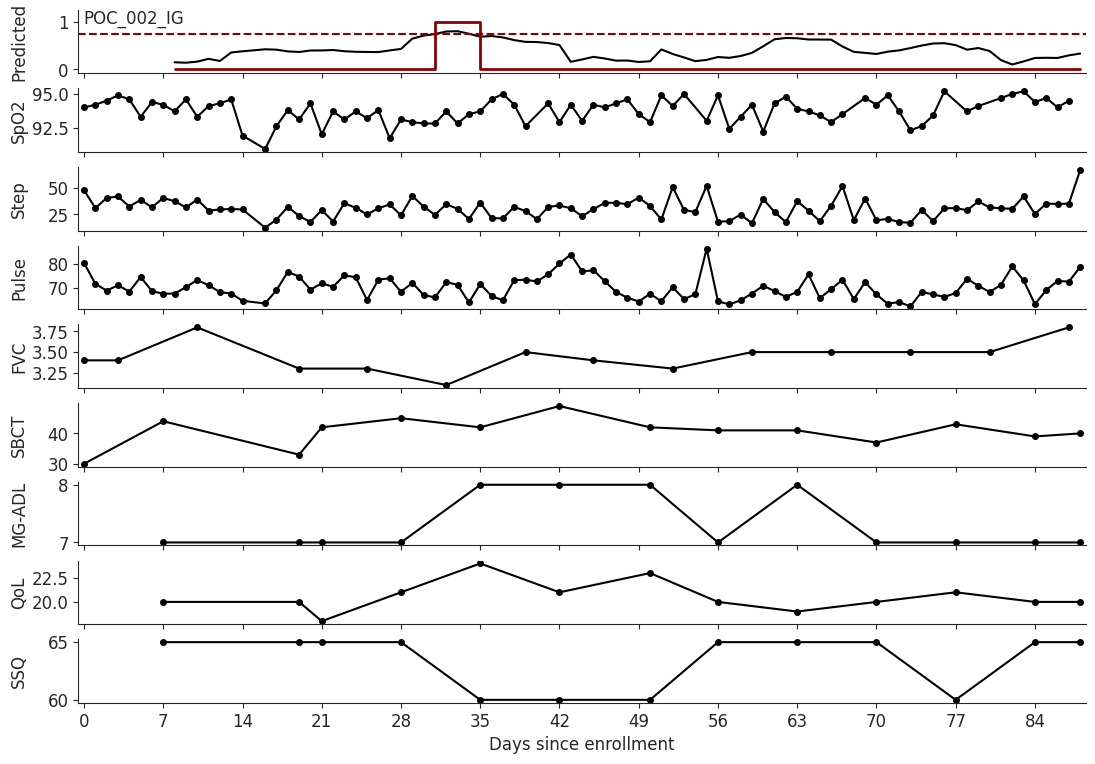


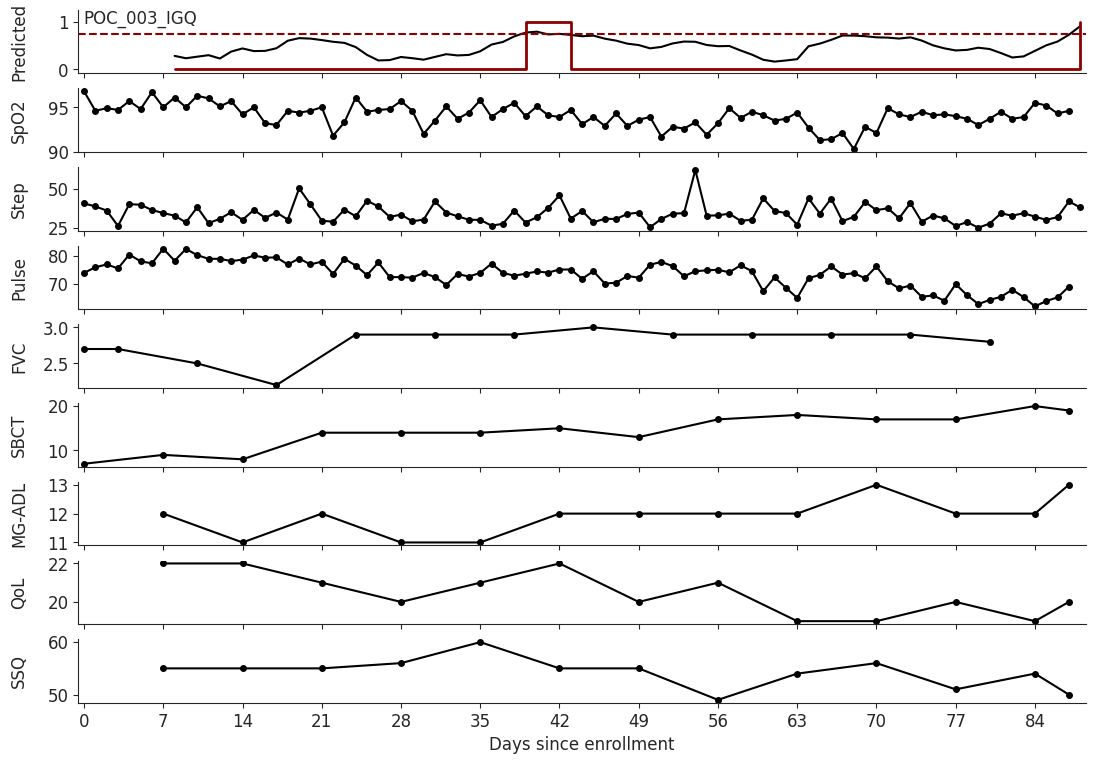

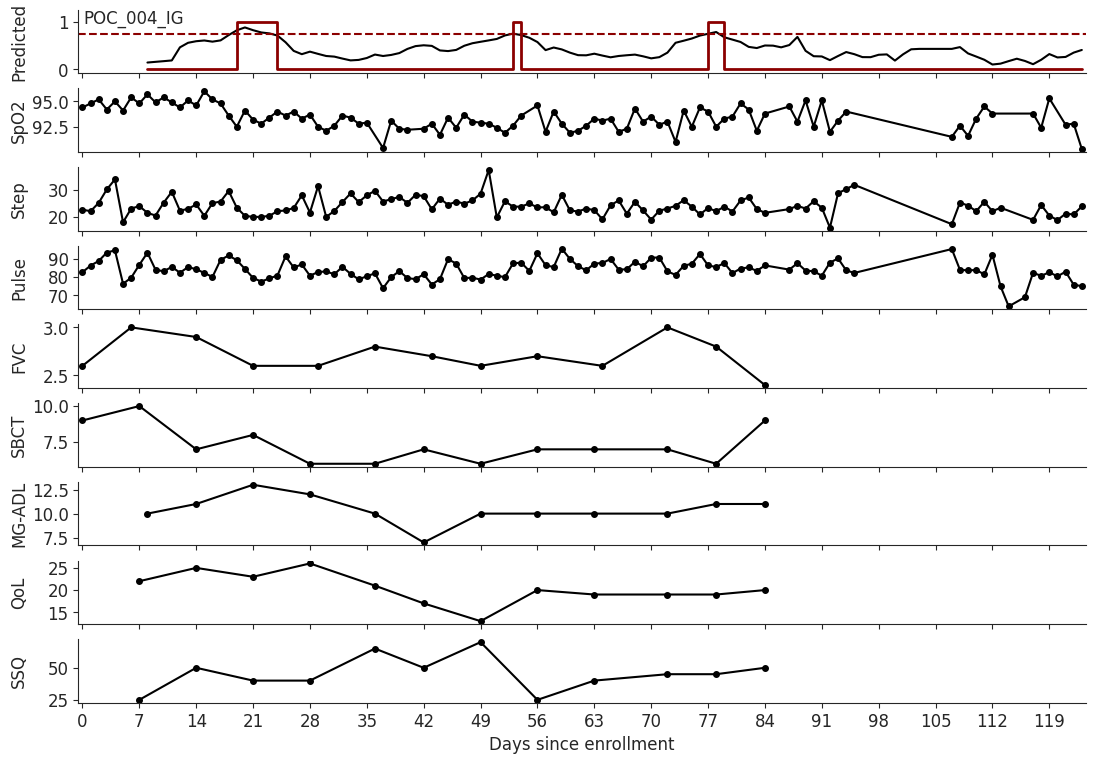

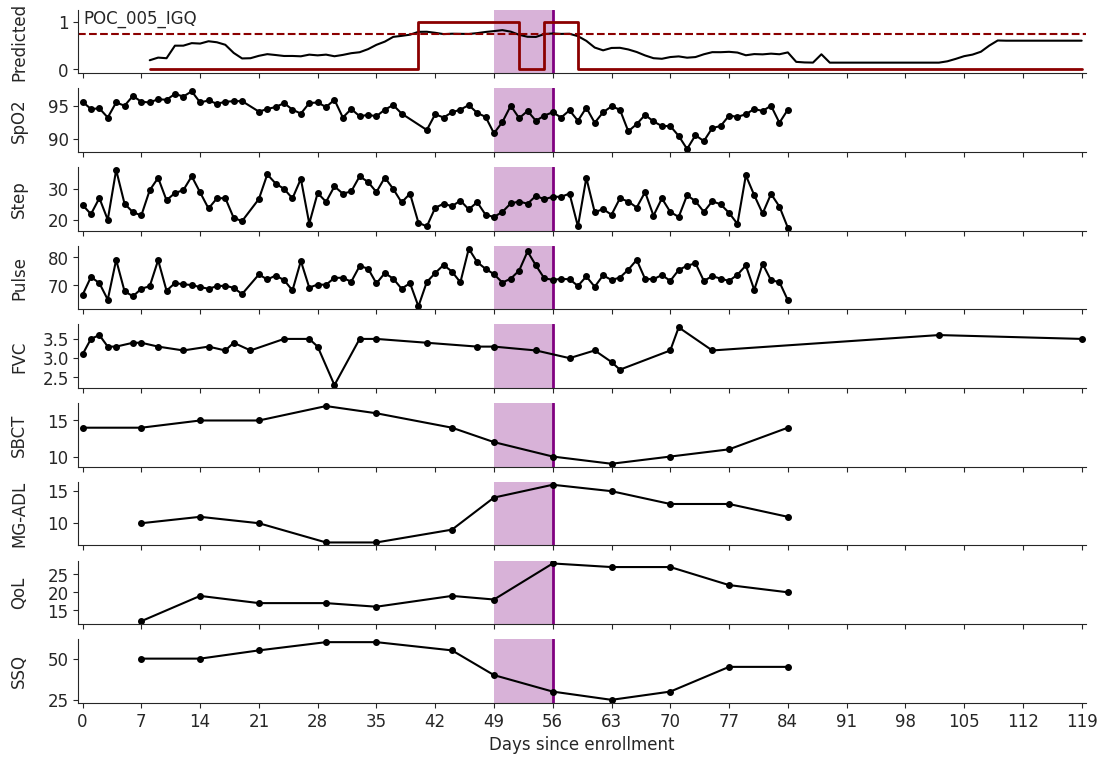

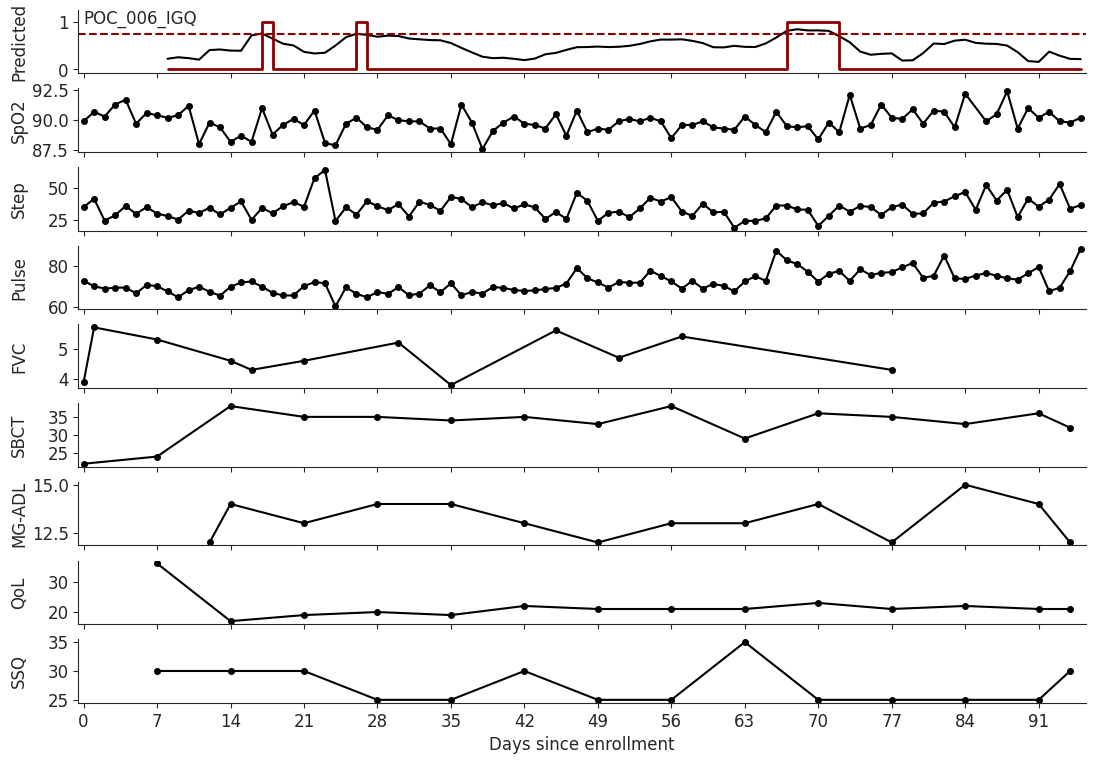

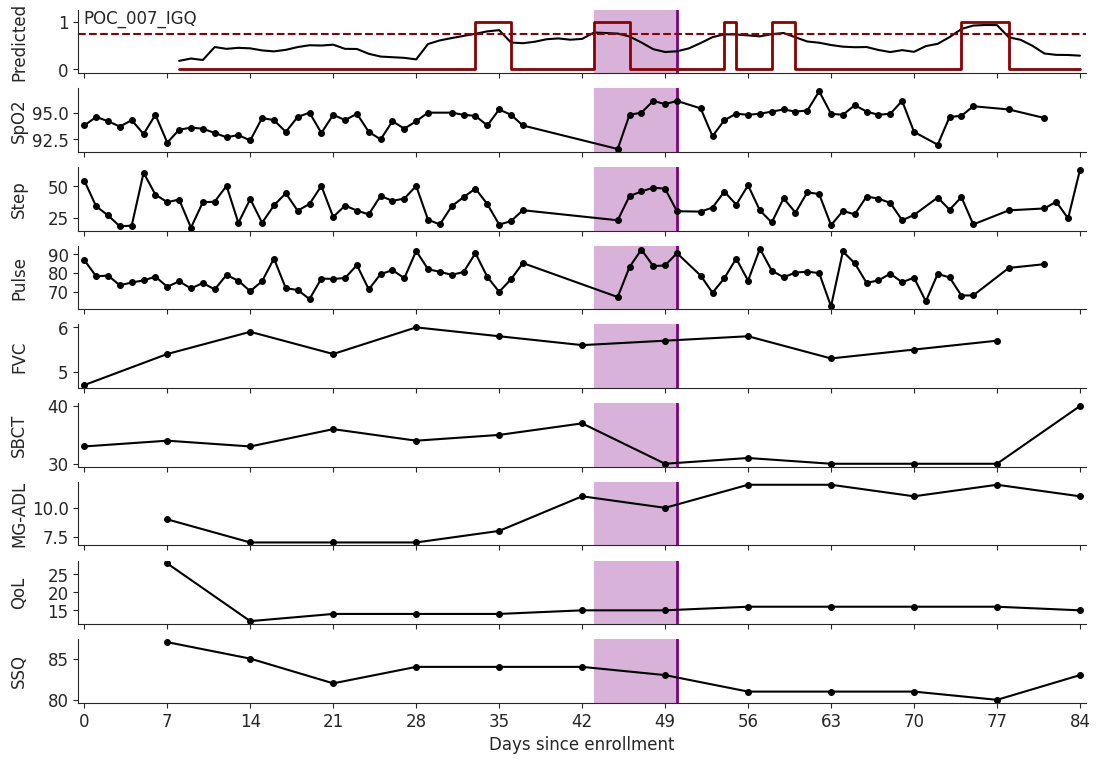

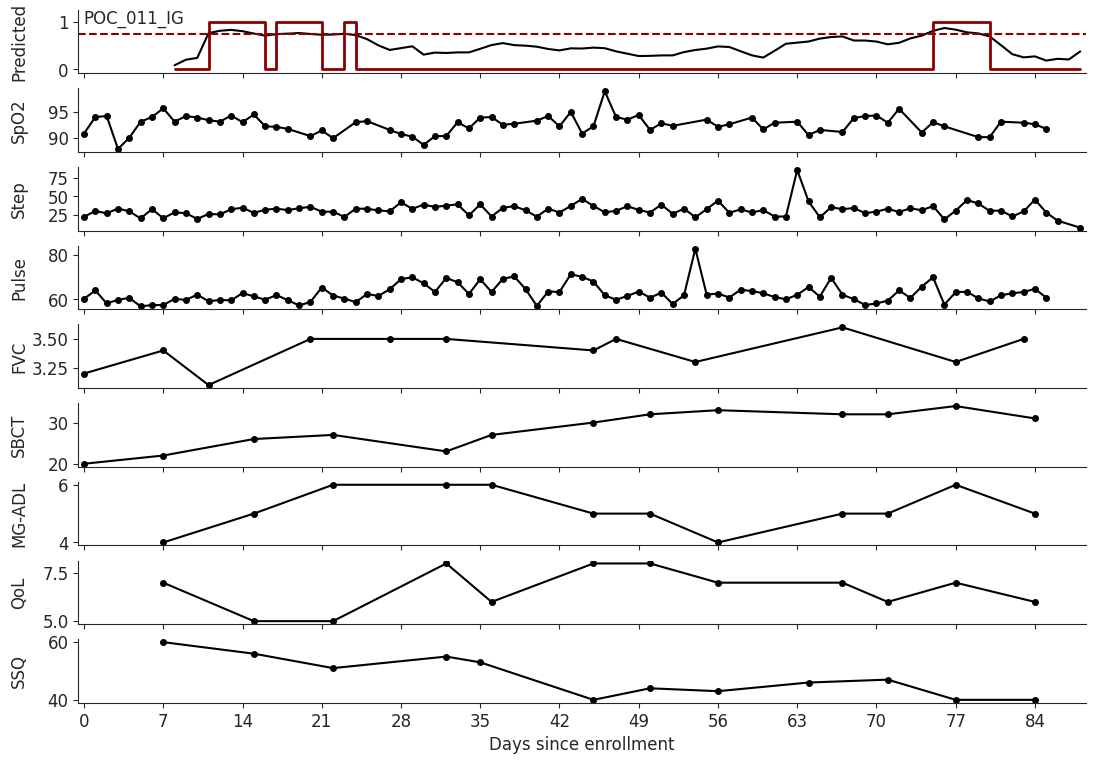

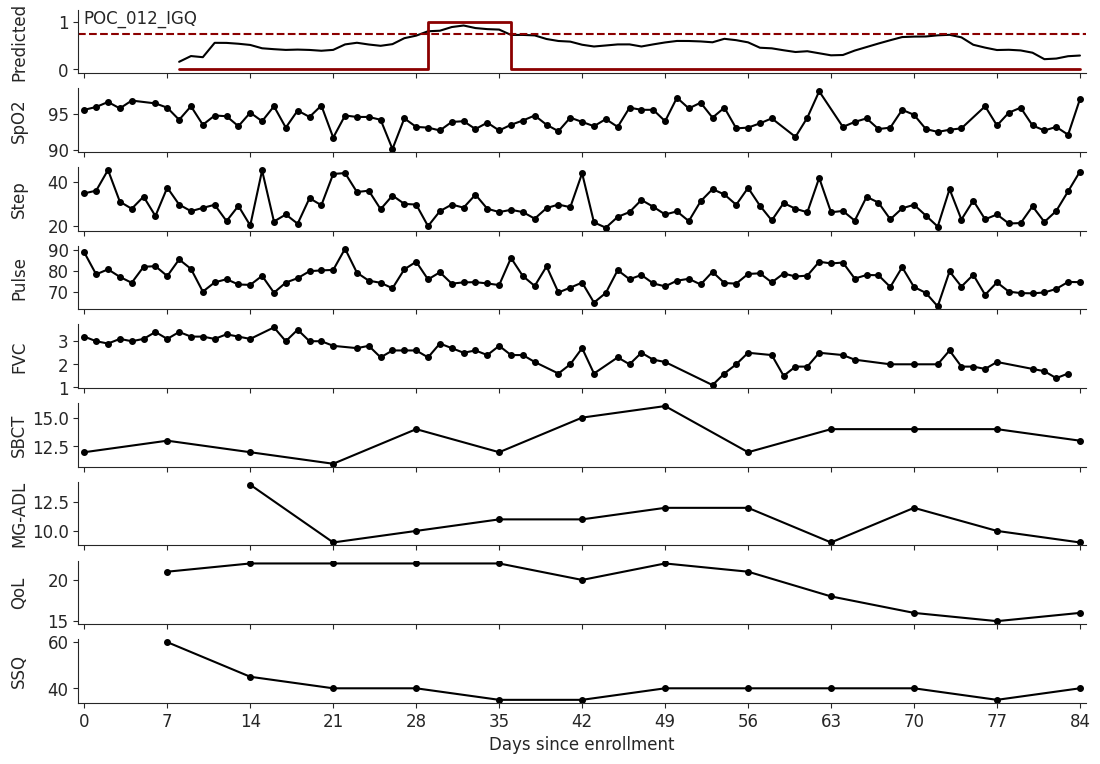

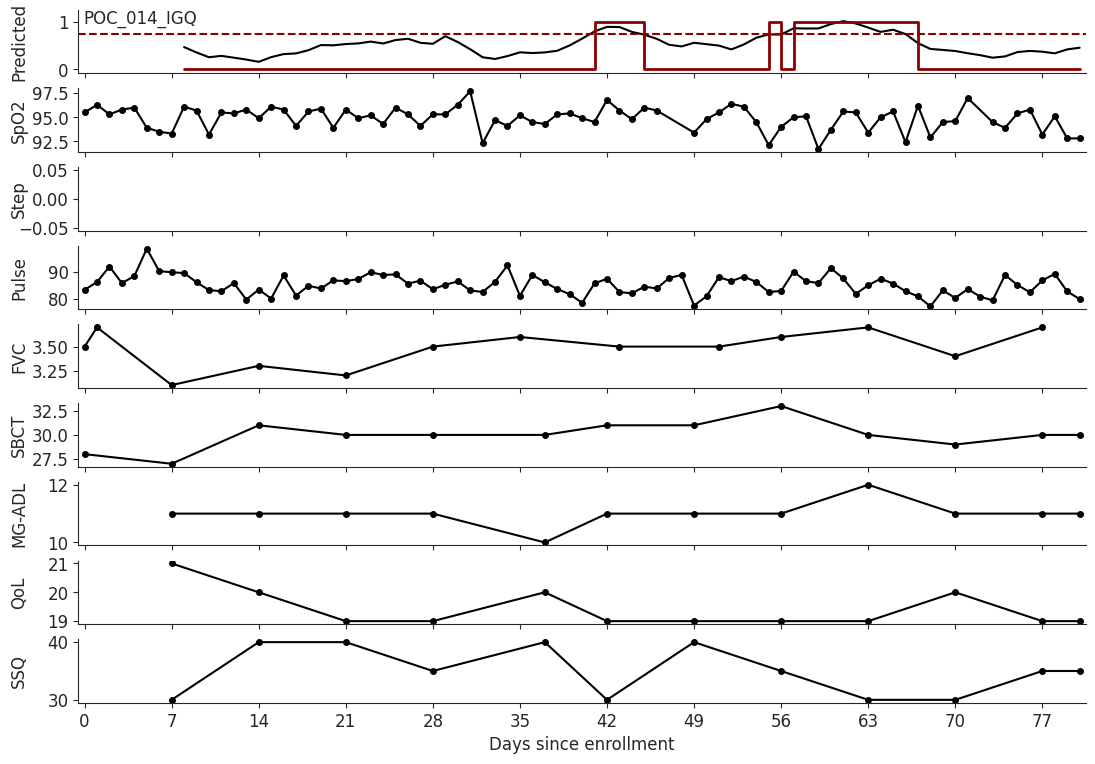

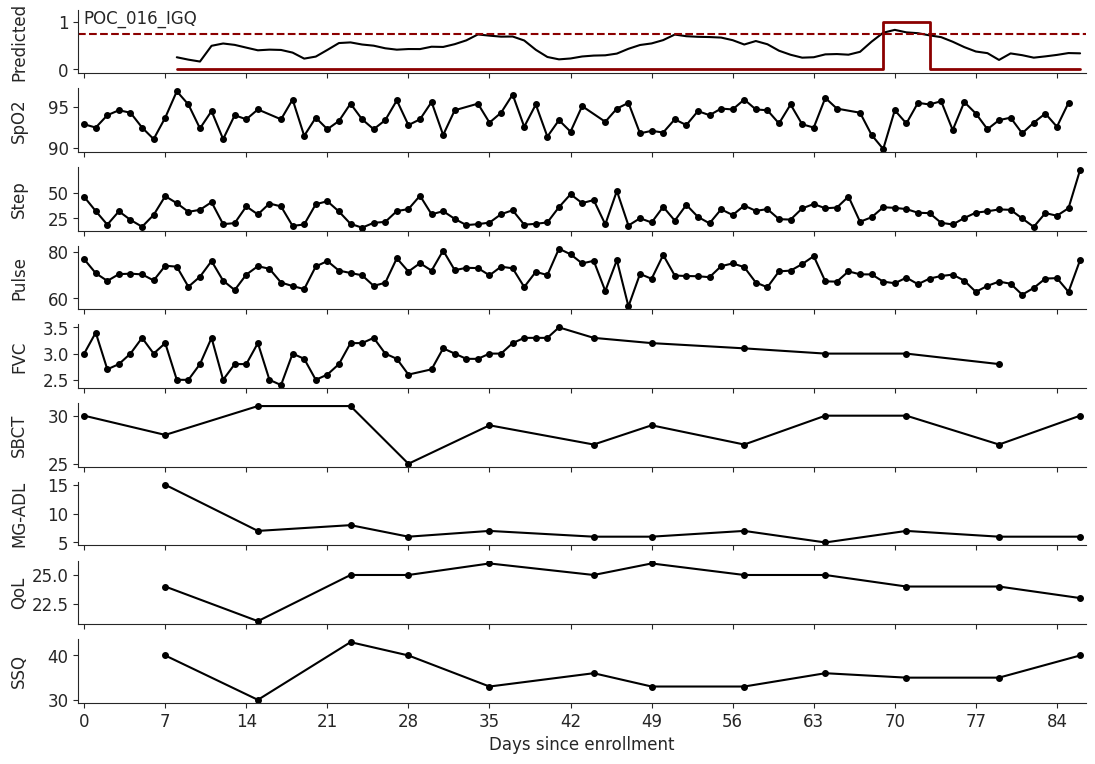

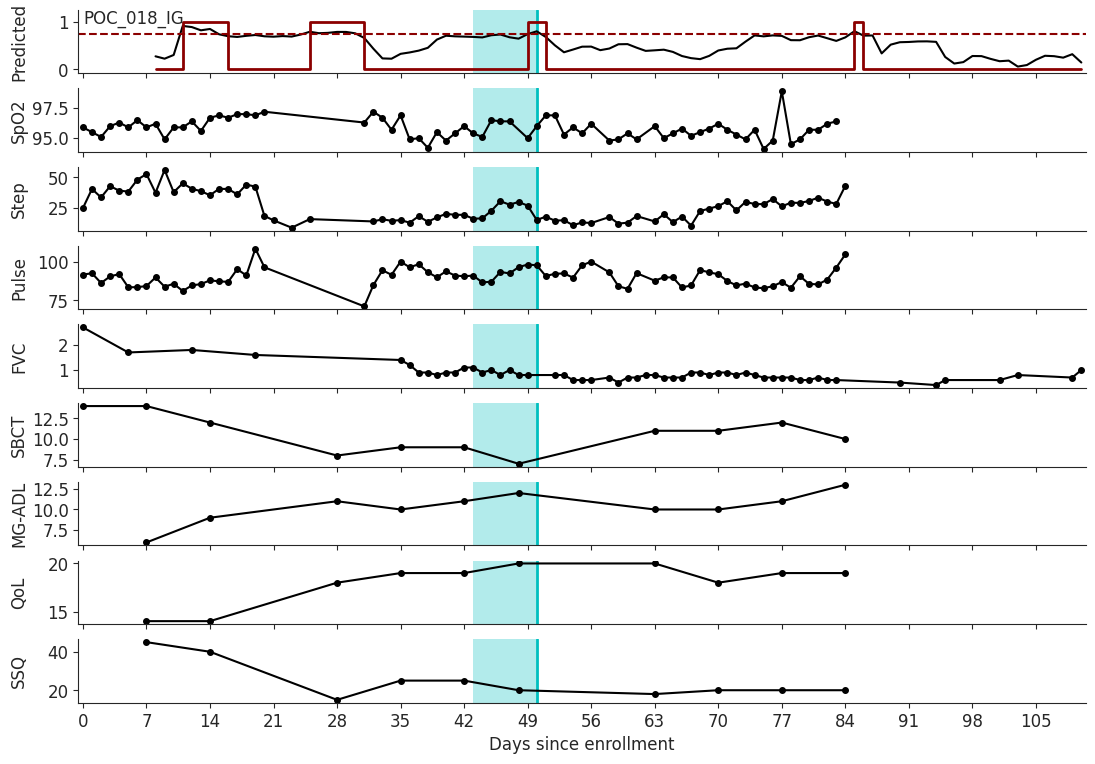

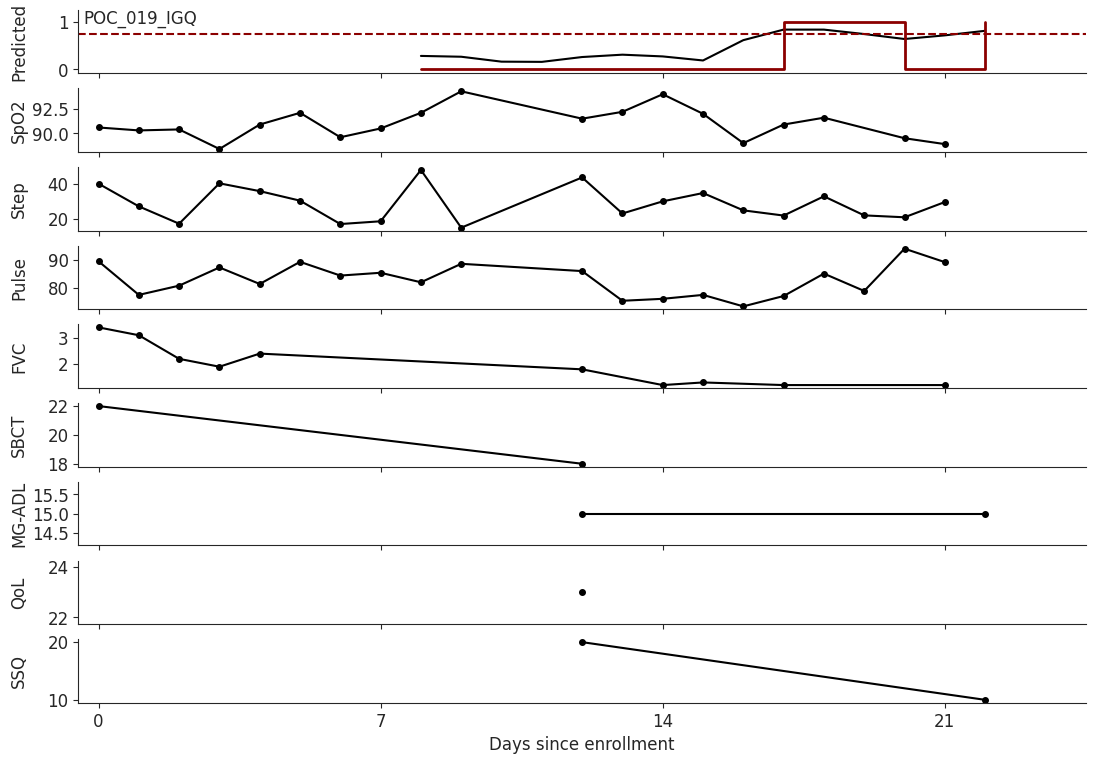

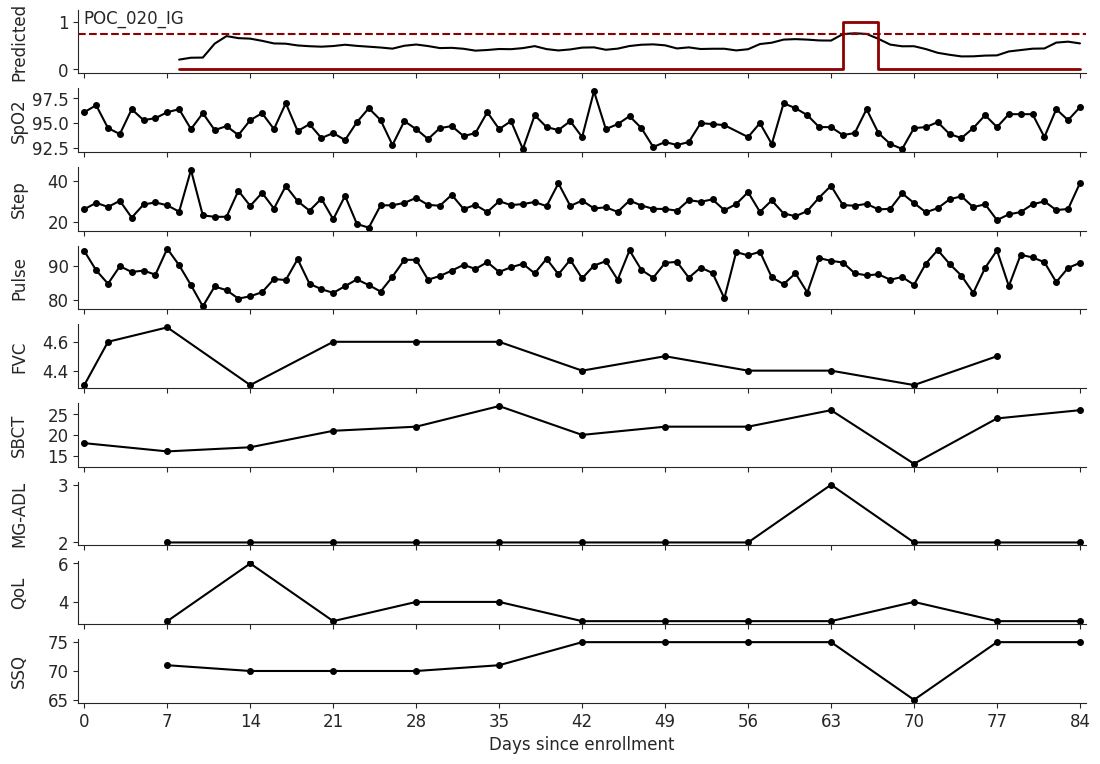

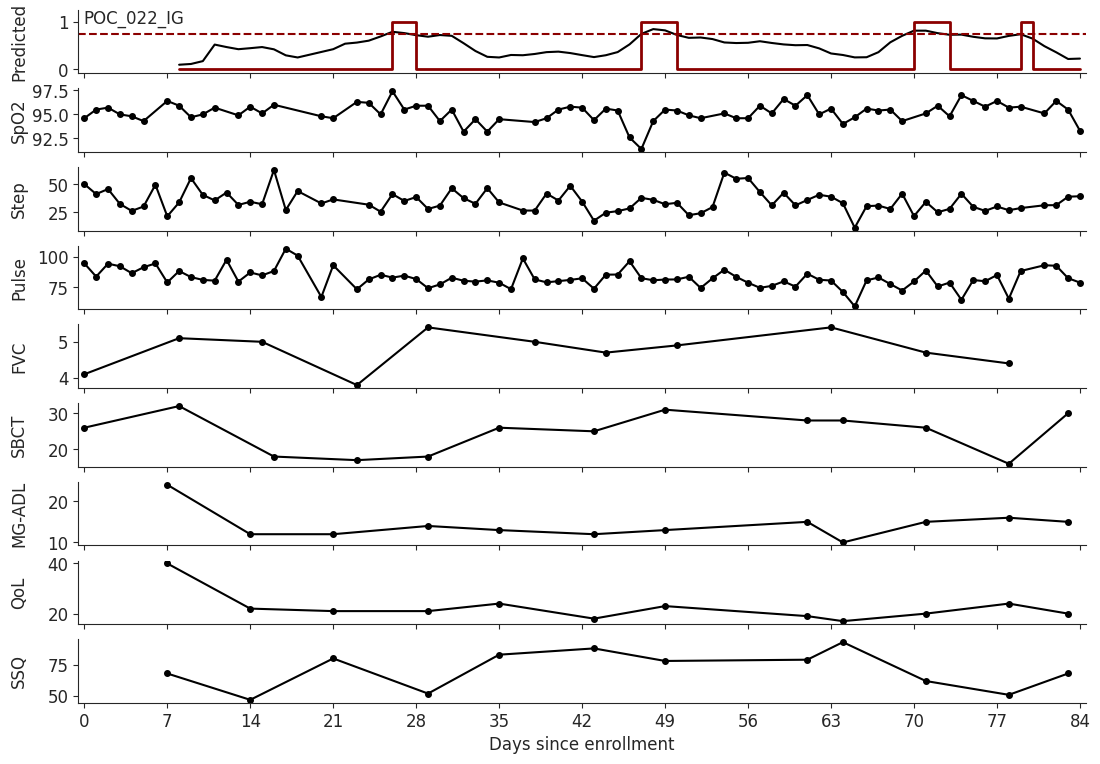

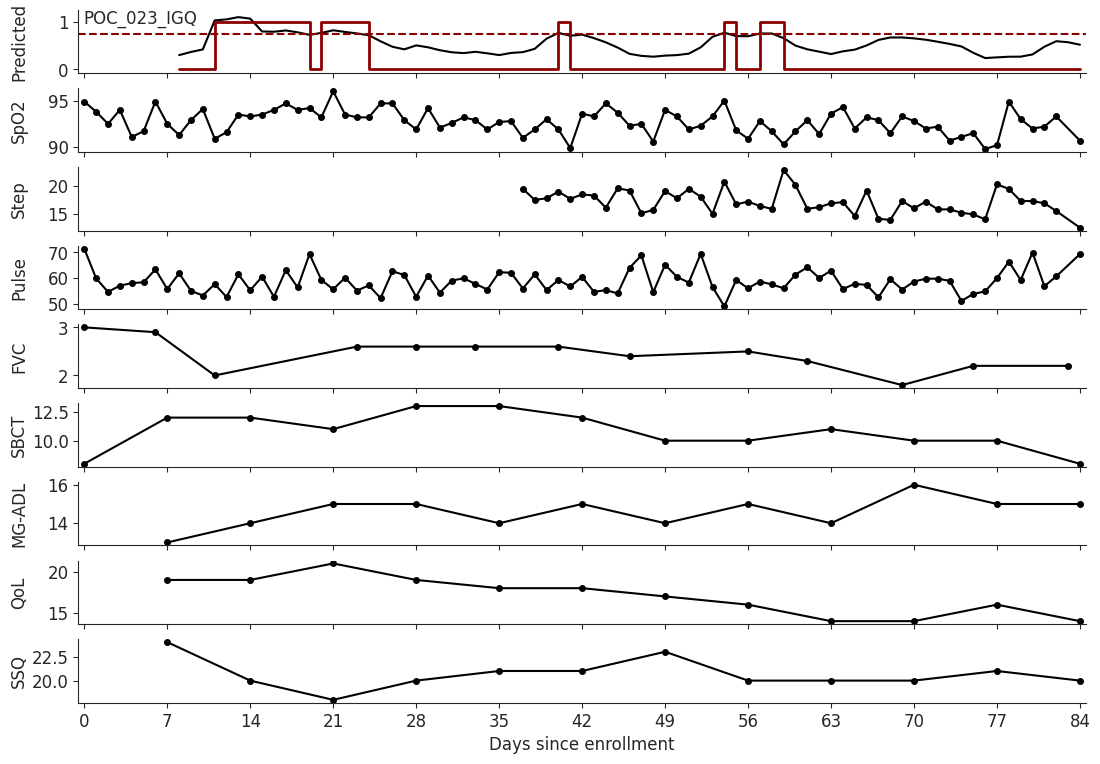

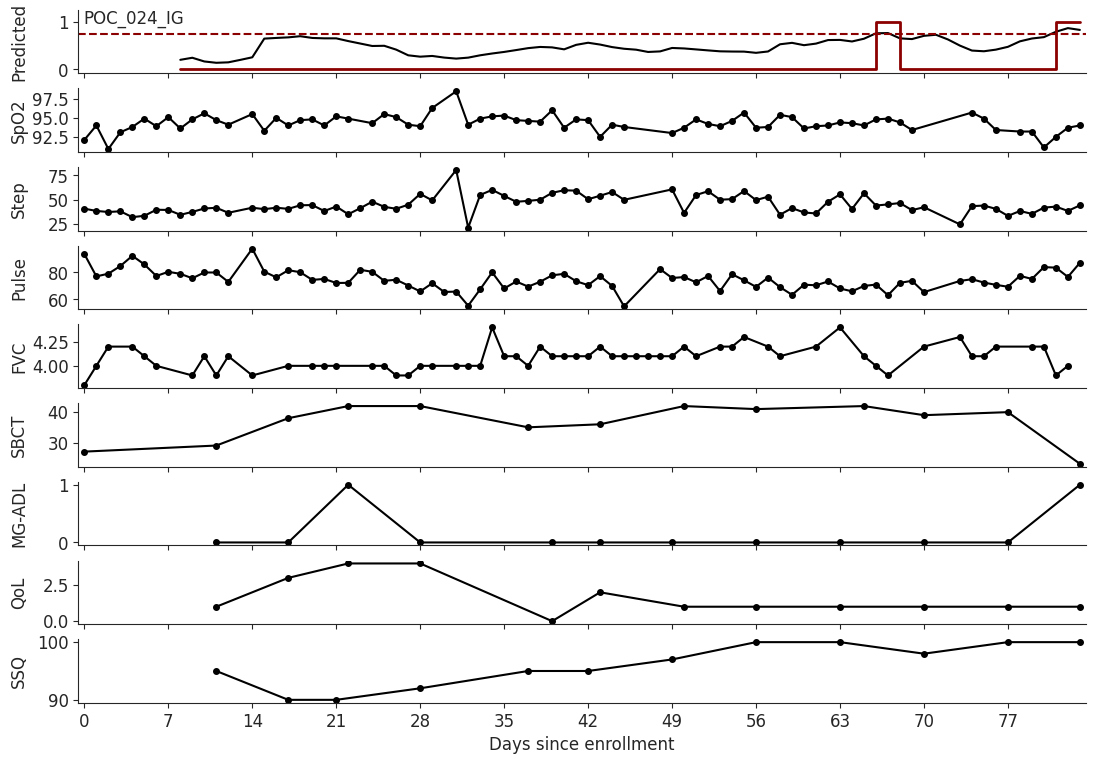

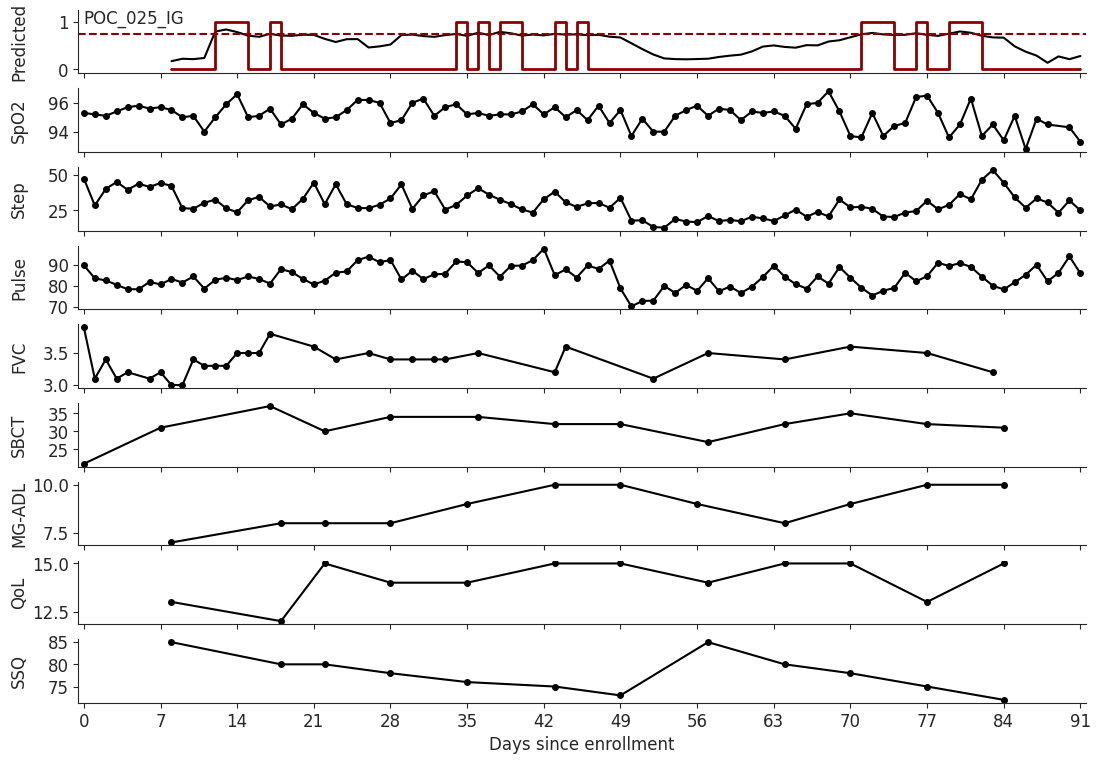

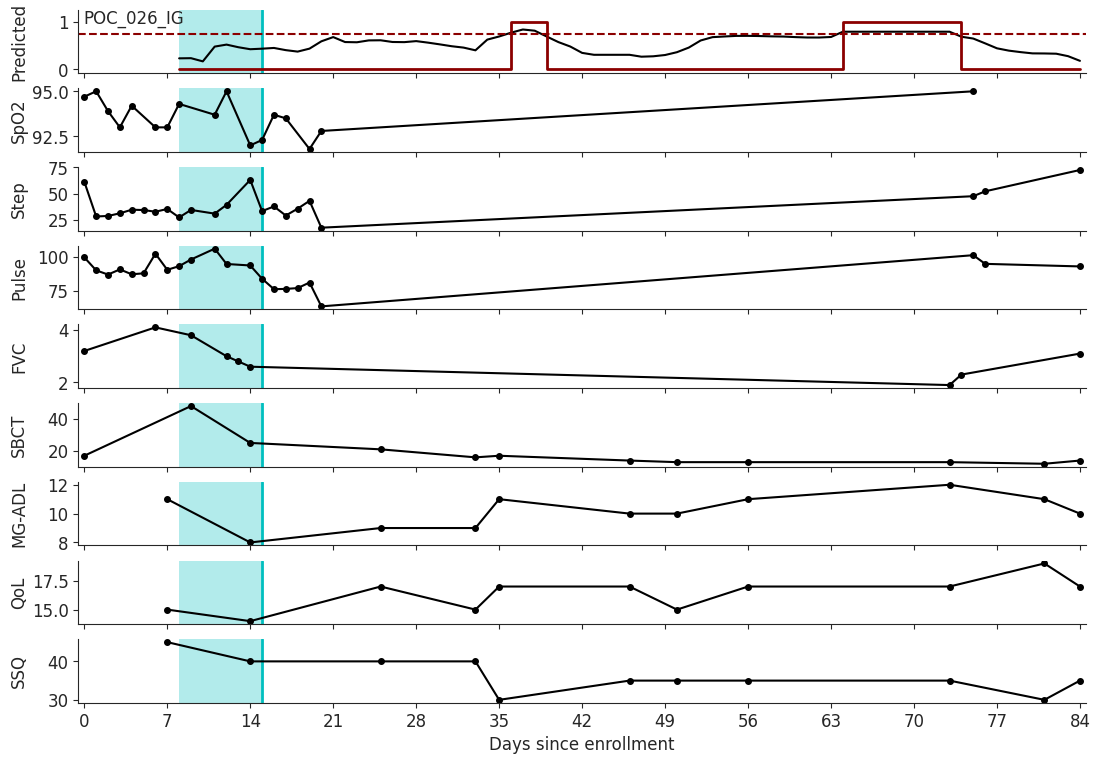

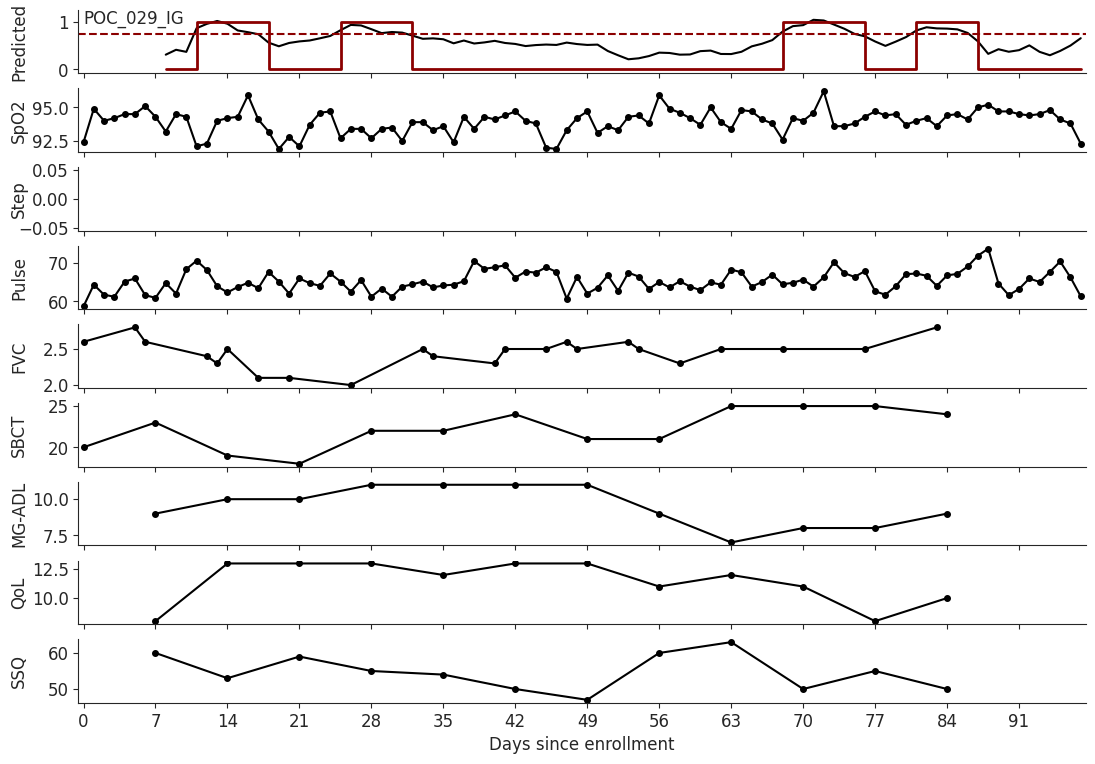

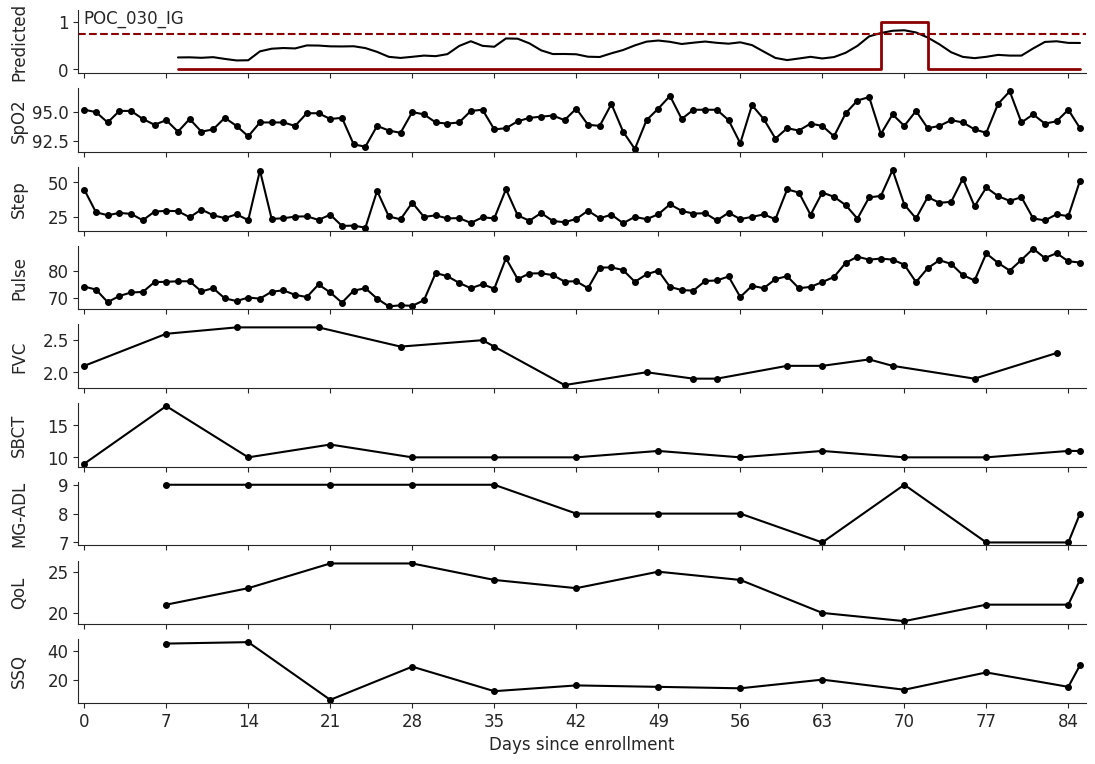

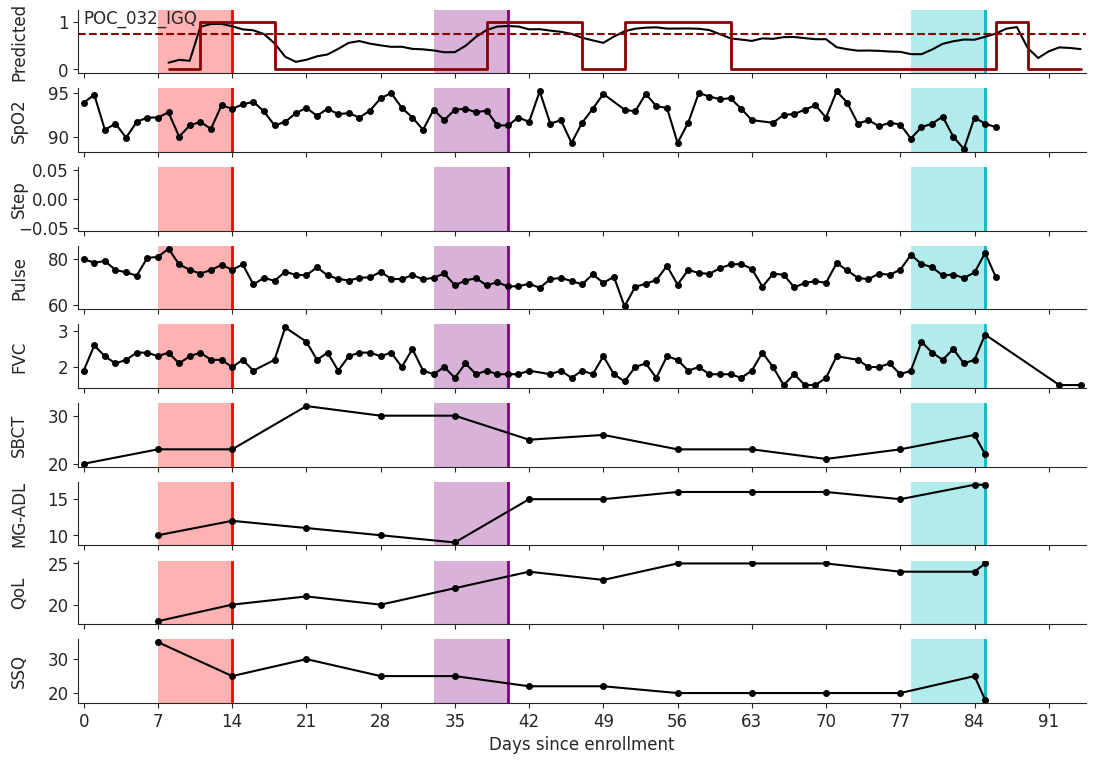

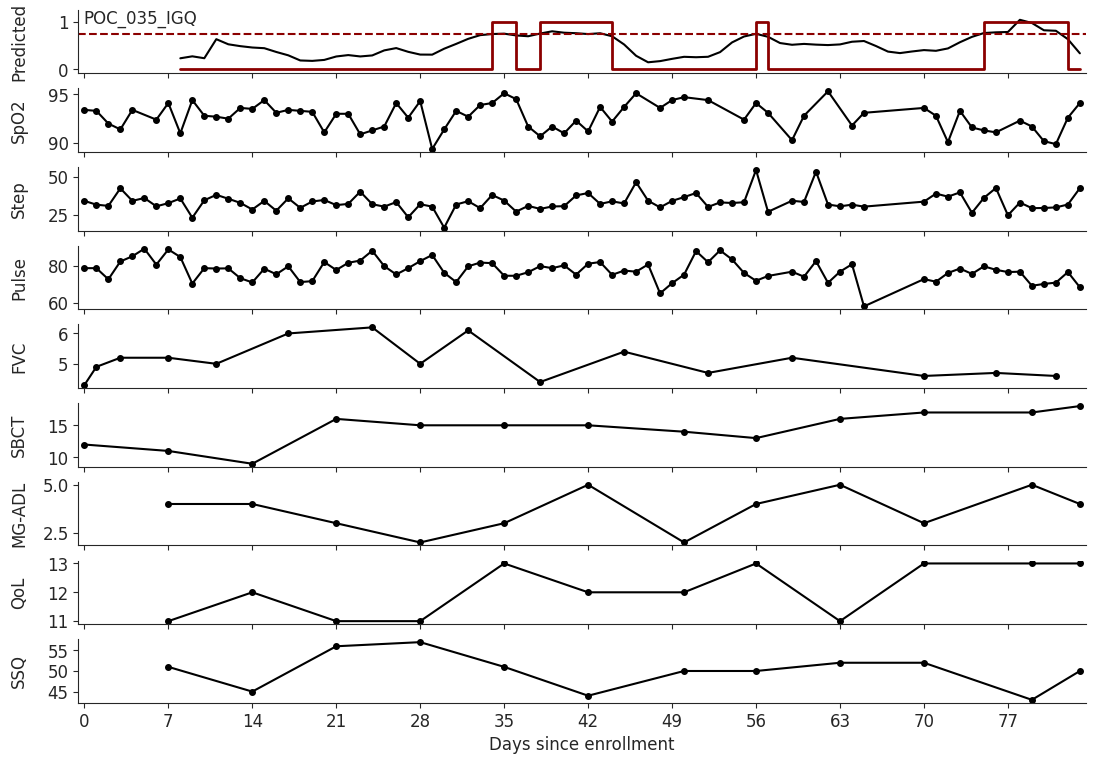

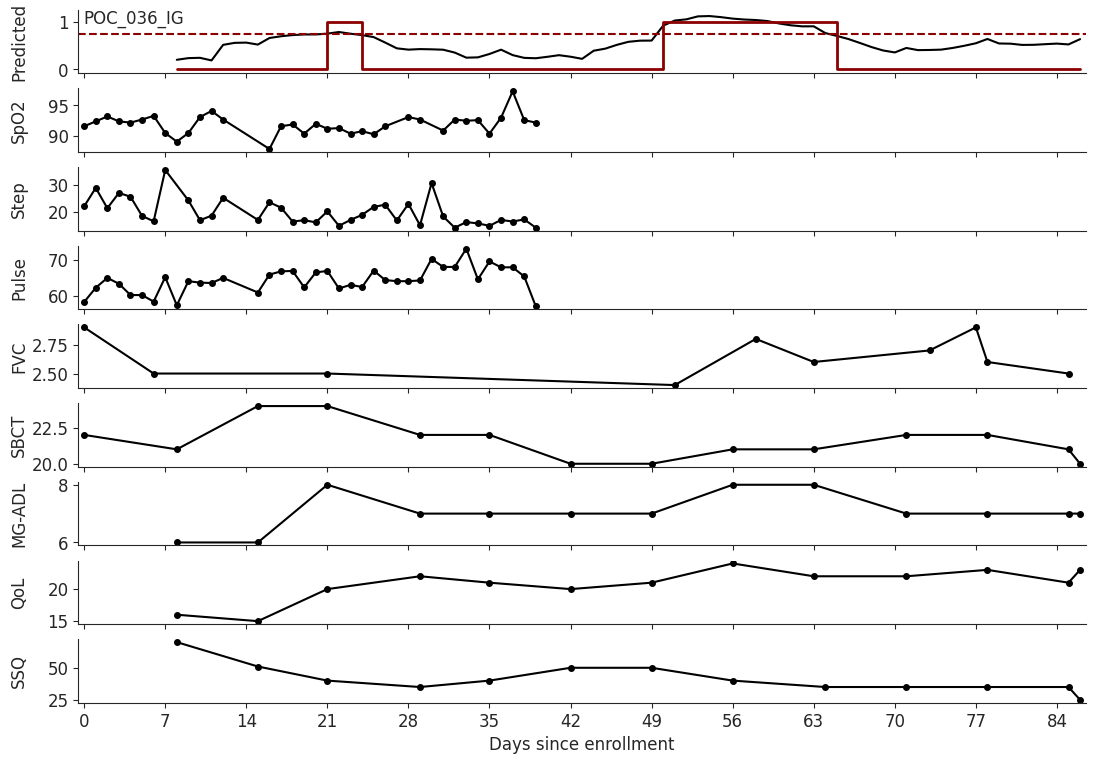

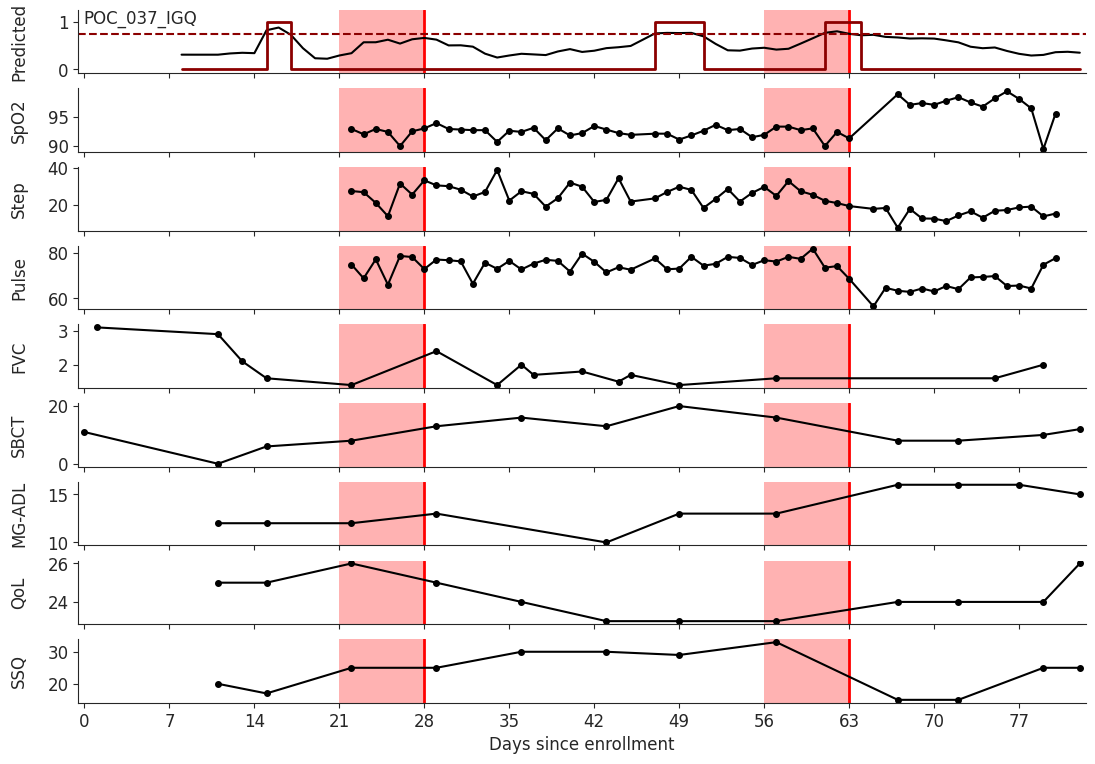

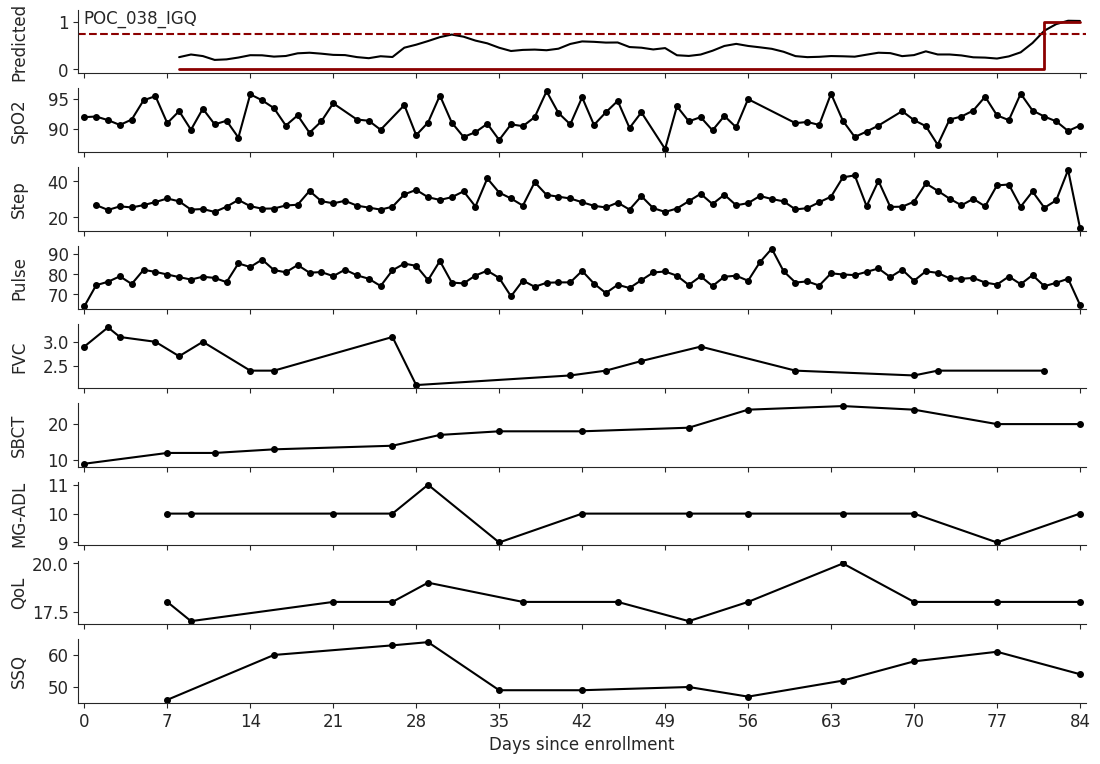

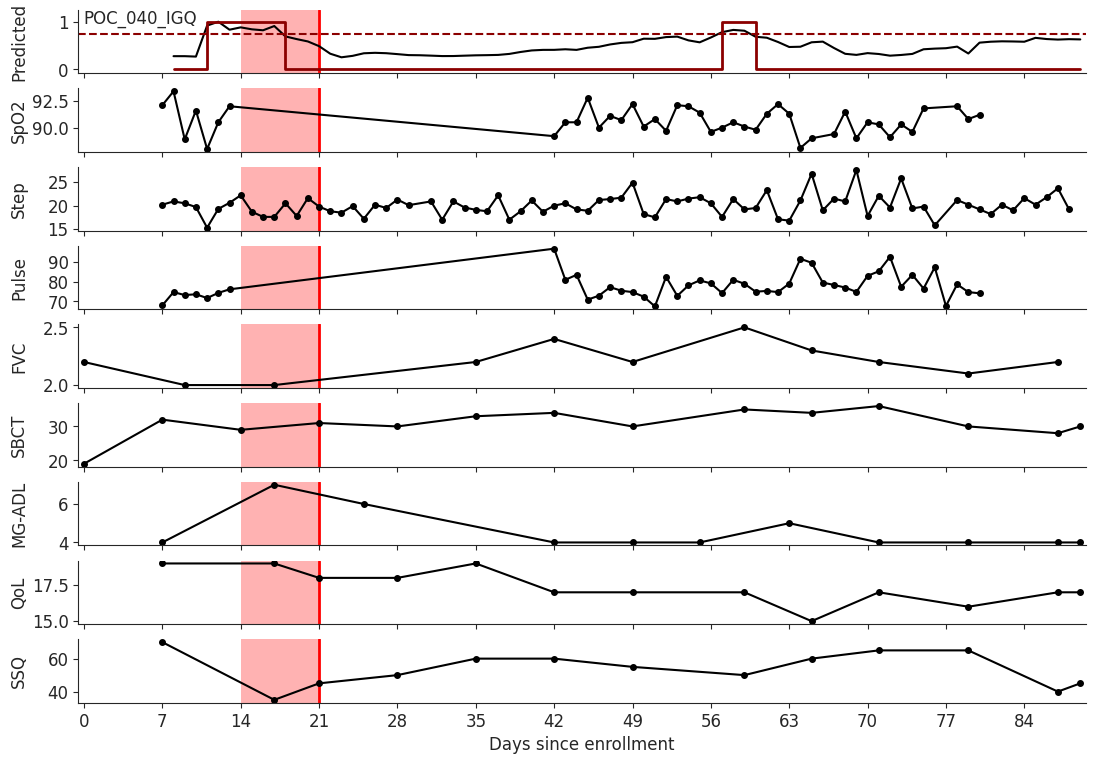

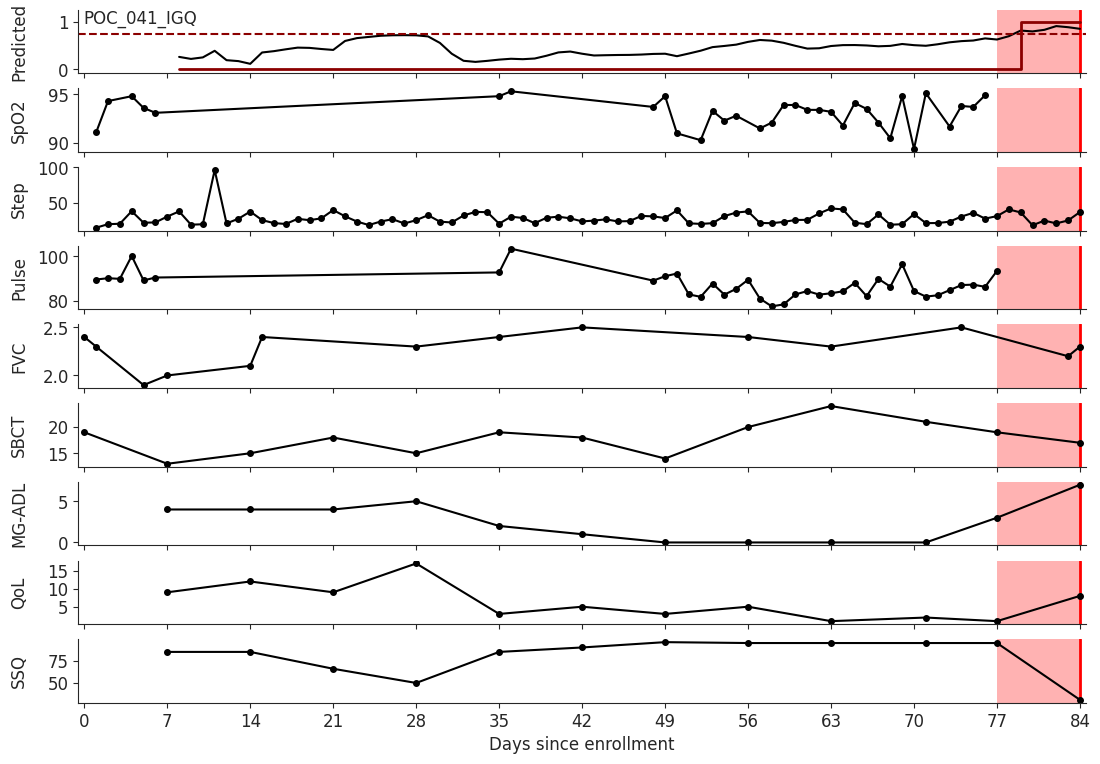

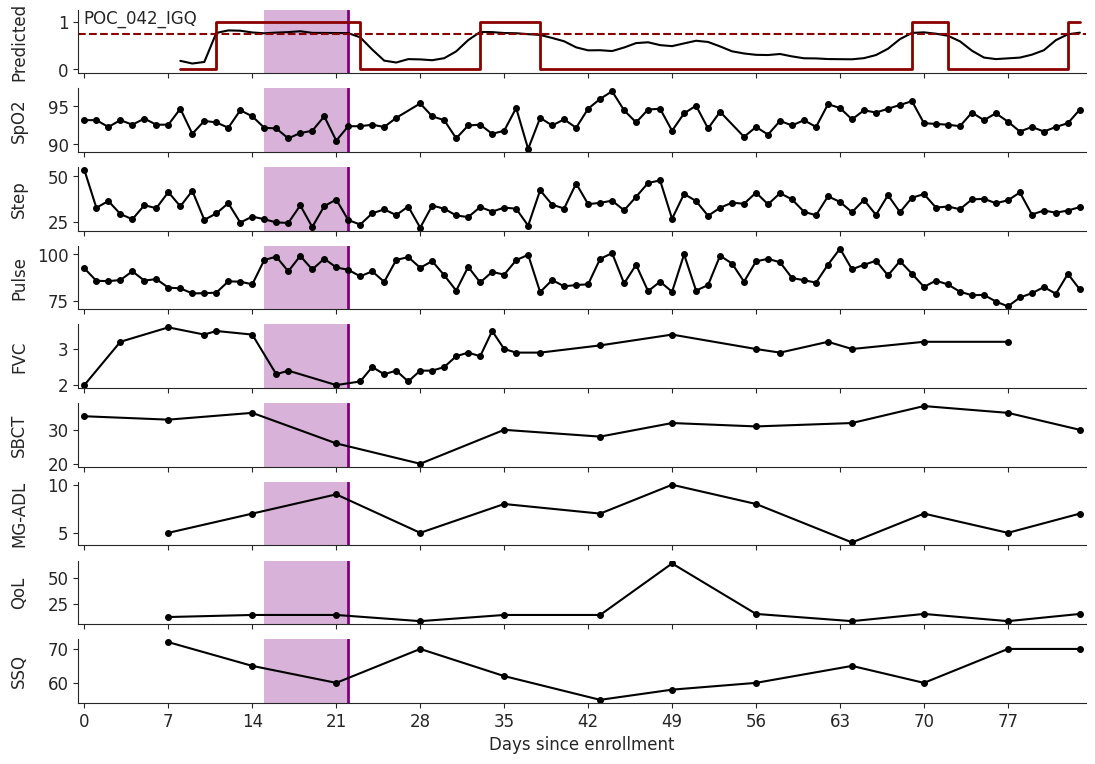

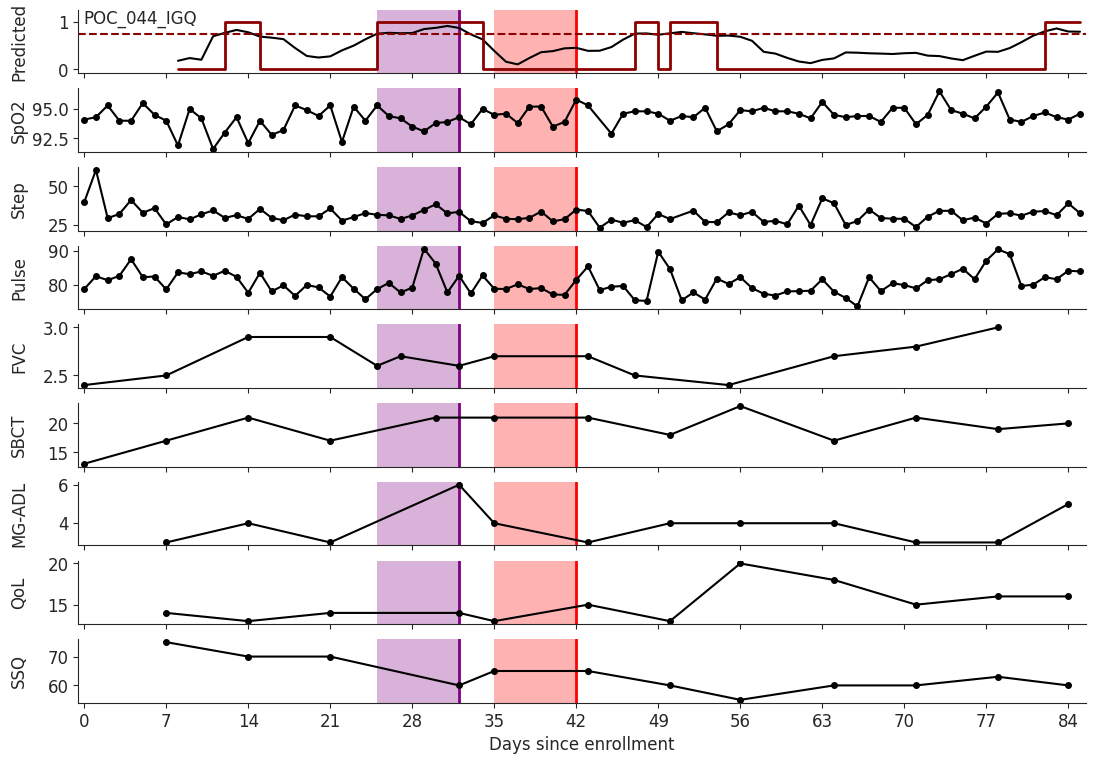

Supplement: Supplementary file 1 — Table S1: Univariate and multivariate AUC and sensitivities. Figure S1: Assessment interval versus performance using univariate wearable signal input. The performances were assessed using a look‐back period (T) of 8 days. Figure S2: Visualization of all cases. The top panel shows the prediction: black curve as the raw prediction, brown binary curve as the binarized prediction, and the horizontal brown dashed line as the threshold for binarization. The vertical lines are deteriorations, with a 7‐day window before the exact day. Red vertical line and window indicate Quantitative Myasthenia gravis score (QMG)‐based deterioration; purple vertical line and window indicate self‐reported deterioration; and cyan vertical line and window indicate hospitalization‐based deterioration. The lower panels indicate the actual signals. Each marker dot represents 1 day. Days without signal are connected using lines. [file ACN3-13-714-s001.docx]
